# Evolutionary games on multilayer networks: coordination and equilibrium selection

**DOI:** 10.1038/s41598-023-38589-6

**Published:** 2023-07-21

**Authors:** Tomasz Raducha, Maxi San Miguel

**Affiliations:** 1 Grupo Interdisciplinar de Sistemas Complejos (GISC), Departamento de Matemáticas, Universidad Carlos III de Madrid, Leganés, Spain; 2 Institute for Cross-Disciplinary Physics and Complex Systems, IFISC (CSIC-UIB), Palma, Spain

**Keywords:** Complex networks, Computational models, Computational science

## Abstract

We study mechanisms of synchronisation, coordination, and equilibrium selection in two-player coordination games on multilayer networks. We investigate three possible update rules: the replicator dynamics (RD), the best response (BR), and the unconditional imitation (UI). Players interact on a two-layer random regular network. The population on each layer plays a different game, with layer I preferring the opposite strategy to layer II. We measure the difference between the two games played on the layers by a difference in payoffs, and the inter-connectedness by a node overlap parameter. We discover a critical value of the overlap below which layers do not synchronise, i.e. they display different levels of coordination. Above this threshold both layers typically coordinate on the same strategy. Surprisingly, there is a symmetry breaking in the selection of equilibrium—for RD and UI there is a phase where only the payoff-dominant equilibrium is selected. It is not observed, however, for BR update rule. Our work is an example of previously observed differences between the update rules. Nonetheless, we took a novel approach with the game being played on two inter-connected layers. As we show, the multilayer structure enhances the abundance of the Pareto-optimal equilibrium in coordination games with imitative update rules.

## Introduction

Spontaneous emergence of coordination between people or animals, without external control, is a remarkable phenomenon that can be crucial for optimal functioning or even survival of the population^1–3^. In some circumstances individuals face making a choice between two or more possible actions, called strategies. It often happens that the best outcome for everyone can be obtained only if we choose the same strategy as our neighbours. In game theory such situation is referred to as coordination game^4–7^. Additionally, it might matter under which strategy the population coordinates. One action can lead to higher prosperity than the other, what is modelled by different strategies having different payoffs. Conditions required to coordinate have been scrutinised under various assumptions and for numerous environments, yet there are still unanswered questions. Here, we study coordination and equilibrium selection in games on multilayer networks.

People interact in multiple contexts and through different media. One natural way to represent it in a strict manner is by using a multilayer network^8–12^. Each layer is a separate network of interactions in a given context. For example, we interact with each other in work place, at home, online etc. In principle, the pattern of interactions can be different in every layer resulting in a different network topology. Additionally, some layers can be hidden^13^. In multilayer networks, if a node exists in many layers, it represents the same person, which often acts similarly in every context. It is therefore connected between layers to itself via inter-layer links, which provide coupling between the layers. It is important to note that, if a system has a multilayer structure, it can not be simply reduced to a single-layer graph without changing the dynamics^14^. Hence, the scrutiny of layered systems is highly relevant.

We use the approach from evolutionary game theory^15–18^ to analyse synchronisation between the layers and equilibrium selection in coordination games, where by synchronisation we mean equal levels of coordination on both layers. Coordination games have been studied in depth on single layer networks, a comprehensive literature review can be found here^19^. From previous results it is worth mentioning the KMR model which explored the equilibrium selection in populations equivalent to complete graphs with the best response update rule^20^. The risk-dominant equilibrium was always evolutionary favoured in the model and several extensions did not find any deviation from this behaviour^21–24^. That outcome is preserved also on a circular network^25^, unless the unconditional imitation is used to update strategies^26^. In general, imitative update rules can favour Pareto-efficiency over risk dominance^27,28^. However, it can only happen in sparse networks—in a complete graph risk-dominant equilibrium is always selected^19^.

Evolutionary games were also extended to multilayer networks^29^. Prisoner’s dilemma was studied on many layers with a possibility of using different strategies on different layers. The strategy was updated according to replicators dynamics, but using the collective payoff from all layers^30,31^. It was also studied together with the stag hunt, the harmony game, and the snow drift on two-layer networks with the game being played on one layer and strategy imitation on the other^32^. Additionally, the same games on one layer were mixed with opinion dynamics and social influence on the second layer^33^. The idea of separating the group in which we play the game from the one where we learn or imitate the strategy had been already studied before within a single network^34–37^. The public goods game^38–40^ was considered on two^41^ and more layers^42^ with the game being played on each layer. Interestingly, in some of the mentioned research the multilayer structure was said to enhance cooperation^30,33,41^. Finally, coordination games were also investigated on multilayer networks. The pure coordination game on one layer was coupled with social dynamics and coevolution on the other, leading to a possible segregation^43^. A particular version of the general coordination game was studied on two interconnected layers, with the strategy being imitated on the layers and the game played between the layers^44–46^. Similarly to single-layer networks, the unconditional imitation and smaller degree favoured the Pareto-optimal equilibrium. However, the body of work on coordination games on multilayer networks is still very limited and consists of particular cases of more complex models mixed with opinion dynamics. Moreover, different works consider different update rules and it is difficult to judge to which extent results are determined by the multilayer structure, the particular payoff matrix, or the chosen update rule. Comparison between different update rules is necessary. For these reasons, we provide a broader analysis of different payoff matrices laying within the coordination games scope together with three different update rules.

We focus on the two-player general coordination game^19^ described by a $2 \times 2$ payoff matrix:1where A and B are available strategies, while *T* and *S* are parameters defining payoffs. By definition, coordination games must fulfil conditions $T<1$ and $S<0$. A game described by such payoff matrix contains a social dilemma. Obviously, the most rewarding outcome is obtained if both players choose the same strategy, but there is a hidden trade off between security and profit. Clearly, the highest possible profit is made when both play the strategy A, hence it is called the payoff-dominant or Pareto-optimal strategy. On the other hand, the risk-dominant strategy is the best choice in the lack of knowledge, i.e. it is the strategy that results in the highest average payoff assuming that the opponent will play either way with the same probability^47^. It is easy to check that for $T<S+1$ the strategy A is risk-dominant, and for $T>S+1$ the strategy B is risk-dominant. This calculation provides a theoretical line $T=S+1$ at which risk dominance changes. When all players coordinate on one of these strategies we refer to such state as a payoff-dominant or risk-dominant equilibrium.

In the evolutionary game theory the game evolves because the players update their strategies after interacting and observing their peers. It is well known that the update rule is as important as the payoff matrix in defining the end result of the game^19,27,28,48–51^. Multiple update rules have been proposed in the literature^52–55^. We focus on three well established ones: the replicator dynamics (RD)^56–58^, the best response (BR)^20,21,25,59–61^, and the unconditional imitation (UI)^17,44–46,62,63^. It is important to note that RD and UI are imitative in nature, as players adapt the strategy of one of the neighbours. BR on the other hand is a strategical update rule which requires from the player knowledge abut the payoff matrix. Another distinction between the update rules is their determinism—BR and UI are deterministic, meaning that the same configuration will always lead to the same strategy being chosen, while RD is a probabilistic update rule. See “Methods” section for more details.

**Figure 1:**
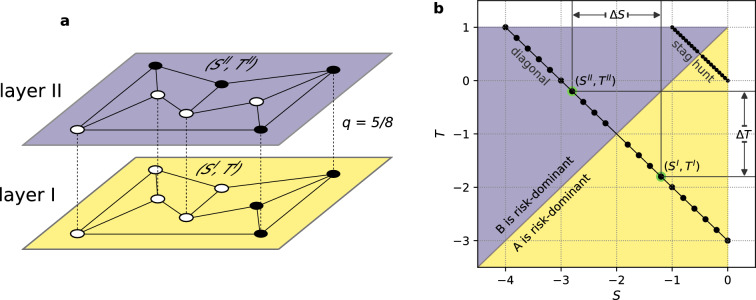
(**a**) Schematic representation of a miniature of multilayer network used in our simulations. Both layers have the same topology of a random regular graph with $N=8$ nodes of degree $k=3$ each and a fraction $q=5/8$ of nodes is shared between the layers. Shared nodes are connected by inter-layer connections (dashed lines). The node overlap *q* is the number of shared nodes divided by *N*. White nodes play the strategy A and black ones play the strategy B. Shared nodes always have the same state on both layers. Each layer has a specific payoff matrix given by $(S^I, T^I)$ and $(S^{II}, T^{II})$. (**b**) Diagram of the *S*-*T* parameter space showing parametrisation of the layers. Each circle on the diagonal lines represents a game played on one of the layers. Examplary values of $(S^I, T^I)$ and $(S^{II}, T^{II})$ are highlighted in green with $\Delta S$ and $\Delta T$ illustrated. On layer I the strategy A is always risk-dominant (yellow area), and on layer II the strategy B is always risk-dominant (purpule area). Risk-dominance changes at the line $T=S+1$.

It was shown that on a single-layer network the risk aversion is usually stronger than the drive to profit. Therefore, on complete graphs the risk-dominant equilibrium is always obtained. For sparse networks under unconditional imitation the system can favour the Pareto-optimal equilibrium over the risk-dominant one, but only for a limited range of parameters^19^. For RD and BR, however, the risk-dominant equilibrium is always selected. In general, local effects were shown to be more important for update rules which have an imitative nature, such as unconditional imitation^26–28^. A natural question is which equilibrium, if any, will be chosen when the population is placed on a multilayer network with two layers on opposite sides of the $T=S+1$ risk-dominance transition line. In other words, on layer I agents play a game where the strategy A is risk-dominant and on layer II a game where the strategy B is risk-dominant. We investigate it by means of numerical simulations.

### The multilayer model

We study a population of players participating in two games on a multilayer network with two inter-connected layers, as depicted in Fig. 1. Both layers have the same number of nodes *N*. If a node is connected to itself between the layers via an inter-link, it plays the same strategy in the two layers. The fraction of nodes connected (or shared) between the layers is controlled by a parameter $q \in [0,1]$, called node overlap or degree of multiplexity^14,64^. There are *Nq* inter-layer connections. For $q=0$ the two layers are effectively two independent networks, for $q=1$ the layers are equivalent to one network (every node has the same state on each layer all the time) playing each game half of the times. The edge overlap^10^ is kept constant and equal to 1 with both layers having the same topology, since we did not observe any change under varying edge overlap. We use random regular graphs^65^. See “Methods” for more details on our simulations.

Players on each layer are engaged in different games, i.e. parameters $S^\beta$ and $T^\beta$, $\beta \in \{ \text {I, II}\}$, defining the payoff matrix have different values on each layer. In order to give the same relevance to both layers, their preferences towards one of the equilibria are set to be equally strong. This is achieved by choosing the points $(S^I, T^I)$ and $(S^{II}, T^{II})$ equally distant from the $T=S+1$ line, as visible in Fig. 1. Another choice to make is the angle between the $T=S+1$ line and the line created by points $(S^I, T^I)$ and $(S^{II}, T^{II})$. We focus on cases where all points lay on a line $T^\beta =-S^\beta +C$, where *C* is a constant (see Supplementary Material for other cases). This is because only then the average payoffs $\langle \Pi ^I \rangle$ and $\langle \Pi ^{II} \rangle$ of both layers are equal, therefore games are truly symmetrical. We analyse the case of $T^\beta =-S^\beta -3$, which we call *diagonal*, and $T^\beta =-S^\beta$ where all games are variants of the well known *stag hunt*^66,67^. Note, that the stag hunt game can be obtained for different values of *C* and that both cases are ,,diagonal” in the sense that they have the same slope. Nevertheless, we call the case of $C=-3$ *diagonal* and $C=0$ *stag hunt* to easily distinguish them in the discussion of results that follows in the manuscript. In both cases we cover with the parameters *S* and *T* the whole width of the general coordination game area (see Fig. 1).

Since the layers are placed symmetrically around the $T=S+1$ line, or more precisely around a point $(S_0, T_0)$ on this line, the parameter $\Delta S = S^I - S^{II}$ is sufficient to determine values of all four parameters $S^I, T^I, S^{II}, T^{II}$. Namely:

2 $$\begin{aligned} {\begin{matrix} &{} S^I = S_0 + \frac{\Delta S}{2} ,\\ &{} T^I = T_0 - \frac{\Delta T}{2} ,\\ &{} S^{II} = S_0 - \frac{\Delta S}{2} ,\\ &{} T^{II} = T_0 + \frac{\Delta T}{2} , \end{matrix}} \end{aligned}$$

where $(S_0, T_0) = (-2,-1)$ for the diagonal case and $(S_0, T_0) = (-0.5,0.5)$ for the stag hunt case. Note also that for $T^\beta =-S^\beta +C$, that is both cases, we have $\Delta S = \Delta T$. From the design of the the system it follows that there is a maximal possible gap size $\Delta S_{max}$ above which the payoff matrices would not describe a coordination game. In Fig. 1 we can clearly see that $\Delta S_{max} = 4$ for the diagonal case and $\Delta S_{max} = 1$ for the stag hunt case.

We use the coordination rate $\alpha \in [0,1]$ to describe the state of the population. When $\alpha ^\beta = 1$ every player on the layer $\beta$ chooses the strategy A, therefore layer $\beta$ is in the Pareto-optimal equilibrium. When $\alpha ^\beta = 0$ the layer also coordinates, but on the strategy B. For $\alpha ^\beta = 0.5$, which is the initial value for each layer, both strategies are mixed in equal amounts in the layer $\beta$. We say that the layers are synchronised when $\alpha ^I = \alpha ^{II}$ and then we use just $\alpha$ to describe both of them. Note that synchronisation does not require coordination within the layers and vice versa, although they usually come together in our results.

**Figure 2:**
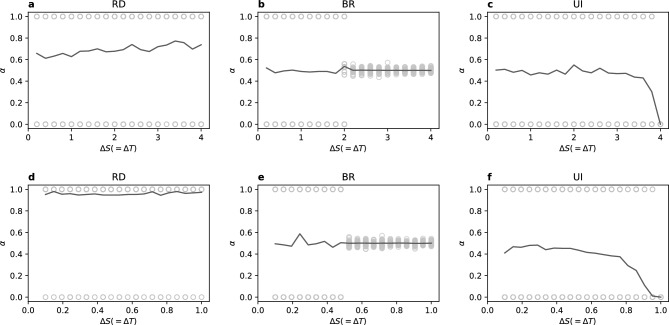
Coordination rate $\alpha =\alpha ^I=\alpha ^{II}$ vs gap size $\Delta S$ for full node overlap $q=1$ (the multiplex case). The upper row (**a**–**c**) presents the diagonal case and the bottom row (**d**–**f**) the stag hunt. For RD and BR each layer has $N=1000$ nodes with an intra-layer degree $k=8$, for UI it is a complete graph with $N=500$. Each circle represents the value of $\alpha$ (for both layers) in one of 400 realisations and solid lines show the average values.

## Results

We study synchronisation between the layers, coordination, and equilibrium selection under varying conditions. For RD and BR update rules we set the connectivity at $k=8$, since it was shown that the degree does not change the equilibrium selection in their case^19^. However, for UI the line $T=S+1$, at which risk-dominance of strategies changes, overlaps with the actual transition in equilibrium selection only for a complete graph^19^. Hence, we analyse the case of unconditional imitation always with full connectivity in order to obtain true symmetry between the layers.

### The effects of game and network parameters

The two main parameters whose influence we investigate are the node overlap *q* and the distance between the games $\Delta S$ or $\Delta T$. For simplicity, we start with an analysis of the multiplex case, i.e. full node overlap $q=1$. In Fig. 2 we present the coordination rate $\alpha$ for synchronised layers at $q=1$ (layers are always synchronised at full node overlap, because all nodes have to be the same on both layers by definition). The first thing to notice is that for the RD update rule the system always coordinates with $\alpha = 0$ or 1 (the circles in the figure). In addition, the RD clearly favours the payoff-dominant strategy A at the maximal level of multiplexity. In the diagonal case the asymmetry is moderate with the average value of $\alpha$ between 0.6 and 0.8 (the solid line in the figure), but in the stag hunt case coordination rarely happens at the strategy B and the average value of $\alpha$ is close to 1.

Like RD, the UI update rule always leads to full coordination in the multiplex case with $\alpha = 0$ or 1. Interestingly, the UI does not favour the strategy A. As we can see in the figure, for small size of the gap $\Delta S$ the outcome is symmetrical with both strategies selected half of the time. But for increasing distance between the pay-off matrices of the two layers the system starts to coordinate more often on the strategy B, to finally select exclusively the non Pareto-optimal equilibrium for the maximal gap size. It has to be noted that the maximal gap size results in payoff matrices that are on the border between coordination games area and non-coordination games, therefore this border point technically does not represent a coordination game. Nonetheless, the decline in the payoff-dominant equilibrium selection is visible already before this limit value. This result is especially surprising, since the UI is the only update rule that on a single-layer network can lead to the Pareto-optimal equilibrium even though it is not risk-dominant^19^. However, the requirement for the selection of a non risk-dominant equilibrium was having a sparse network and here the UI update rule is analysed on a complete graph.

The only truly symmetric update rule is the BR which does not reveal any preference towards one of the strategies for full node overlap. Additionally, the diagonal case is identical to the stag hunt case. For gap sizes $\Delta S < \Delta S_{max} / 2$ and $q=1$ synchronised layers reach either equilibrium with equal probability, and for $\Delta S > \Delta S_{max} / 2$ the system does not coordinate staying at $\alpha = 0.5$. At the transition value of $\Delta S = \Delta S_{max} / 2$ both states—the coordination on one of the strategies and non-coordinated fully-mixing state—are possible (see Fig. 2).

**Figure 3:**
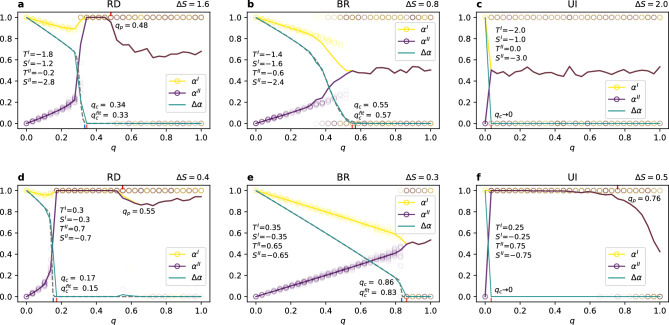
Coordination rates on layers $\alpha ^{I}$, $\alpha ^{II}$, and $\Delta \alpha$ vs node overlap *q* for exemplary values of $\Delta S$ (see Supplementary Material for other values). The upper row (**a**–**c**) presents the diagonal case and the bottom row (**d**–**f**) the stag hunt. For RD and BR each layer has $N=1000$ nodes with an intra-layer degree $k=8$, for UI it is a complete graph with $N=500$. Each circle represents one of 500 realisations and solid lines show the average values. For each realisation there is one circle for layer I (yellow) and one for layer II (purple). Note, that when layers synchronise $\alpha ^{I} = \alpha ^{II}$, $\Delta \alpha = 0$, and both circles overlap looking like one of brownish colour, as well as the solid lines for $\alpha ^{I}$ and $\alpha ^{II}$ merge (brown). The dashed line in (a, b, d, e) shows a function fitted to $\Delta \alpha$.

In addition to the results showed in Fig. 2 for $q=1$, we know that for $q=0$ each layer will obtain full coordination on its preferred strategy—A for layer I and B for layer II^19^. The middle ground between those two extreme values of *q* must therefore contain some kind of transition. We investigate it in Fig. 3, where we can see how the coordination rate $\alpha$ changes at both layers with increasing *q*. The values of $\Delta S$ in the figure were chosen as good examples of general behaviour for each update rule, see Supplementary Material for other values. First thing to notice is that for any update rule and any parameter choice, but with $q=0$, each layer converges to a different limit value of $\alpha$. This means that both layers indeed obtain full coordination on their preferred strategies, as expected for separate networks. Consequently, the difference between layers is maximal $\Delta \alpha = 1$ and each network selects the risk-dominant equilibrium. Similarly, for $q=1$ layers must fully overlap with $\Delta \alpha = 0$, as observed in Fig. 3, because each node is present on all layers and the state of a shared node must be the same across the layers.

### The critical value of node overlap

The above considerations lead to a conclusion that there must be a certain point $q_c \in [0,1]$ at which $\Delta \alpha$ becomes zero. In Fig. 3 we see that the value of $q_c$ can vary for replicator dynamics and best response update rules, but is close to zero for unconditional imitation. In fact, $q_c \rightarrow 0$ for any configuration of the layers when players update their strategies according to UI (see Supplementary Materials for plots of different cases). In other words, synchronisation between the layers is the strongest for the UI update rule. One has to still bear in mind that for UI we have considered a complete graph, while for RD and BR the networks are much sparser with $k=8$. Nevertheless, simulations for higher degree for BR indicate that synchronisation is weakened, not strengthened, by increasing connectivity (see Supplementary Materials), which makes the update rule a natural explanation of the observed differences.

Another surprising observation is that not all the results are symmetrically placed around $\alpha = 0.5$. Both layers have equally strong preferences towards their natural equilibria—payoff matrix parameters $(S^I, T^I)$ and $(S^{II}, T^{II})$ are equally distant from the transition line $T=S+1$ and average payoffs of the games on both layers are the same. There is no reason, in principle, why the system as a whole should choose one equilibrium over the other. Nevertheless, we can see that for some parameters’ values with RD and UI synchronised layers coordinate exclusively on the Pareto-optimal strategy A ($\alpha = 1$), while it does not happen for the strategy B at any point (except for $q=1$ with the maximal gap $\Delta S$ for UI, see Fig. 2). This symmetry breaking is especially interesting, because it is driven by the level of multiplexity *q* in a non-trivial way. In examples shown in Fig. 3, and in general, if the Pareto-optimal equilibrium is obtained on both layers it happens as soon as they synchronise, i.e. at $q_c$. When increasing the node overlap further at some point $q_p$ the synchronised state with coordination on the strategy *B* starts to appear and the average value of $\alpha$ drops below 1. For $q>q_p$ synchronised layers can coordinate on either strategy, however $\alpha >0.5$ in most cases meaning that the Pareto-optimal equilibrium is still dominant. It is important to note that sometimes $q_c=q_p$ and the system goes directly from no synchronisation to coordination on either of the strategies. This is the case visible in Fig. 3b,c,e, where indeed there is no pure Pareto regime.

**Figure 4:**
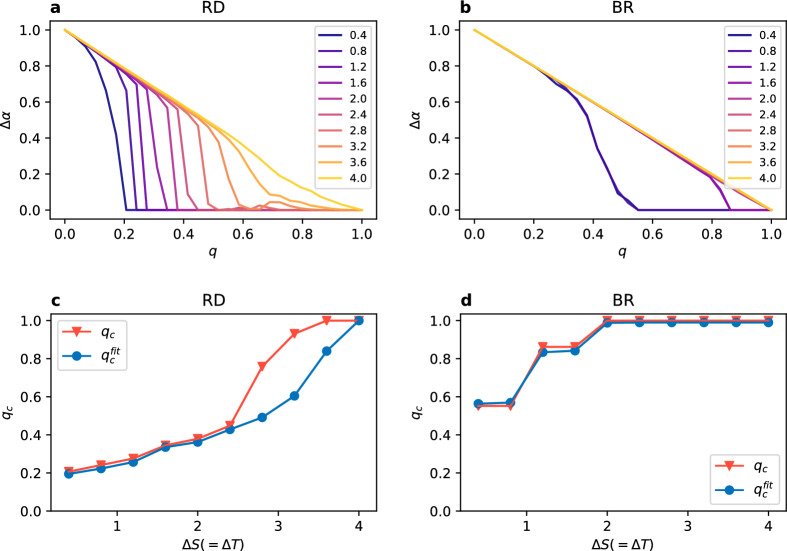
(**a**,**b**) Coordination rate difference between the layers $\Delta \alpha$ vs node overlap *q* for increasing value of $\Delta S$ (given in the legend). (**c**,**d**) Critical value of $q_c$ and $q_c^{fit}$ vs gap size $\Delta S$. Results for the diagonal case, $N=1000$ nodes on each layer with an intra-layer degree $k=8$ averaged over 100 realisations.

**Figure 5:**
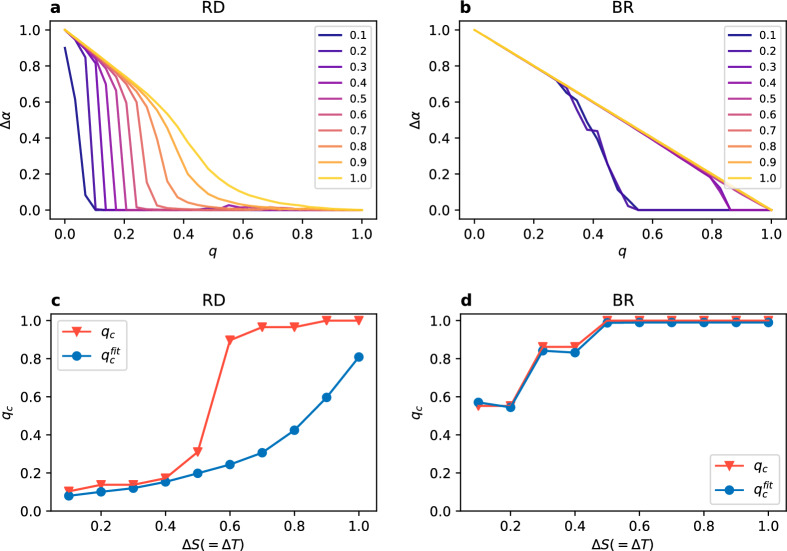
(**a**,**b**) Coordination rate difference between the layers $\Delta \alpha$ vs node overlap *q* for increasing value of $\Delta S$ (given in the legend). (**c**,**d**) Critical value of $q_c$ and $q_c^{fit}$ vs gap size $\Delta S$. Results for the stag hunt case, $N=1000$ nodes on each layer with an intra-layer degree $k=8$ averaged over 100 realisations.

The fully symmetrical outcome that one would expect from the symmetrical design of the experiment is obtained solely for BR. We can see in Fig. 3 that there are only two types of behaviour that the system displays with BR update rule. The first one, for $q<q_c$, is characterised by no synchronisation between the layers and each of them following a specific level of coordination, which is $\alpha ^I = -q/2+1$ and $\alpha ^{II} = q/2$. This calculation comes from a simple assumption that all nodes that are not shared play the dominant strategy of their layer and all shared nodes play either strategy half of the time. Put differently, half of the shared nodes play the strategy A and half the strategy B. This gives a fraction $(1-q)+q/2=-q/2+1$ of nodes playing the strategy A on the layer I and the same fraction of nodes playing the strategy B on layer II, so $1-(-q/2+1)$ of them playing the strategy A. As we can see in the figure this scenario is realised until reaching $q_c$. The second type of behaviour, for $q>q_c$, is coordination of both synchronised layers on one of the strategies with equal probability of choosing either of them.

The behaviour observed so far leads to a question about the change, if any, we would observe when varying the distance between the layers, i.e. for different values of the gap size $\Delta S$. In Figs. 4a,b and 5a,b we present the dependence of $\Delta \alpha$ on the degree of multiplexity *q* for values of $\Delta S$ ranging from 0.4 to 4 in the diagonal case, and from 0.1 to 1 for the stag hunt. This range essentially covers the whole width of the general coordination game area, as presented in Fig. 1. What we can see is that $\Delta \alpha$ drops to zero at higher node overlap when increasing the gap size. More precisely, for RD it roughly follows the line of $\Delta \alpha = -q +1$ to diverge from it at some point and eventually reach the lowest possible value of 0. The line is followed for much longer in the diagonal case than in the stag hunt case. For BR there is virtually no difference between those cases and the dependence on the gap size is slightly different. Values of $\Delta \alpha$ are the same for gap sizes equal 0.4 and 0.8, then again for 1.2 and 1.6, and from $\Delta S = 2$ onwards $\Delta \alpha = -q +1$ (these values are for the diagonal case, for stag hunt the general picture is the same with values rescaled by a factor of 1/4).

**Figure 6:**
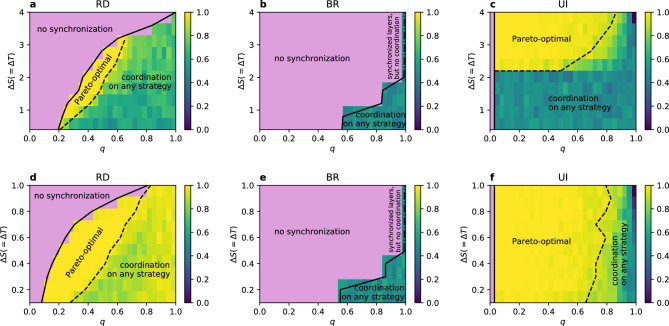
Phase diagram of coordination rate $\alpha =\alpha ^I=\alpha ^{II}$ in the *q*-$\Delta S$ space, for synchronised layers for the diagonal (**a**–**c**) and the stag hunt (**d**–**f**) case. The pink area represents the range of parameters where synchronisation is not obtained and $\alpha ^I \ne \alpha ^{II}$ (for UI it happens only at $q=0$). The solid lines show the critical value $q_c^{fit}$ and the dashed lines $q_p$. For RD and BR each layer has $N=1000$ nodes with an intra-layer degree $k=8$, for UI it is a complete graph with $N=500$. Results are averaged over 100 realisations.

We can clearly see that $q_c$ depends on the gap size $\Delta S$ and this dependence is presented in Figs. 4c,d and 5c,d. We use two approaches in order to estimate the value of $q_c$. The first one is simply taking the lowest value of *q* at which $\Delta \alpha$ is equal 0 for the first time. This approach, however, is prone to numerical noise and a tiny divergence from 0 will result in a change of the value. To obtain the second one we fit a parabola with an exponential cutoff to the function $\Delta \alpha (q)$ (dashed line in Fig. 3) and we take the first value of *q* at which $\Delta \alpha < 0.01$ as $q_c^{fit}$. As we can see in the plots, it does not make a real difference for BR, but can give different results for RD for higher values of $\Delta S$. Regardless the approach, $q_c$ changes from approximately 0.2 up to 1 for RD in the diagonal case (for the stag hunt values are slightly lower), and from 0.5 to 1 for BR with no visible difference between the diagonal and the stag hunt case. Note, that we don’t present the value of $q_c$ for UI in a plot, because it is constant ($q_c \approx 0$) regardless of the parameter choice. We also estimate the value of $q_p$, however without fitting a function, because the behaviour of $\alpha$ for synchronised layers is more complex than the one of $\Delta \alpha$. We take as an approximation of $q_p$ the first value of *q* after synchronisation for which the coordination rate $\alpha$ drops below 0.95 (dashed lines in Fig. 6).

### The final phase diagram

In summary, for any gap size $\Delta S$ (or $\Delta T$) between the layers at $q=0$ there is no synchronisation and each layer gravitates towards its preferred equilibrium. Then, at $q=q_c$ layers start to synchronise. For RD and UI synchronised layers coordinate on the Pareto-optimal strategy for $q_c<q<q_p$ and for $q>q_p$ they coordinate on either of the strategies. For some values of $\Delta S$, however, as well as for BR in general, $q_p$ overlaps with $q_c$ and the system goes from unsynchronised state straight into coordination on any strategy, without the phase of pure Pareto-optimal equilibrium. We illustrate all these results with phase diagrams in the *q*-$\Delta S$ space in Fig. 6. Additionally, there are two update-rule-specific phenomena. For UI at the maximal gap between the layers ($\Delta S_{max} = 4$ for the diagonal case and $\Delta S_{max} = 1$ for the stag hunt) and for $q=1$ synchronised layers coordinate only on the strategy B just at this point. And for BR for $\Delta S > \Delta S_{max} / 2$ at full node overlap when the layers get synchronised they do not reach coordination. Instead they both end up in a fully mixing state with $\alpha ^I=\alpha ^{II}=0.5$ (see panels b and e of Fig. 2 and of Fig. 6).

We can also see from Fig. 6 that an increase in the absolute values of payoffs $S^\beta$ and $T^\beta$ on both layers, i.e. a shift from the diagonal to the stag hunt case, significantly enlarges the relative area of Pareto-optimal equilibrium for RD and UI. It does not, however, change the relative size of the no-synchronisation phase and it seems not to influence the best response dynamics at all. One explanation of the enlargement of the Pareto-optimal phase, at least for RD, could be the fact that in the stag hunt case the layers are closer to each other—the gap size $\Delta S$ (and $\Delta T$) is 4 times smaller on average. Games being more similar and closer to the transition line could justify why it is easier for layer I to shift layer II into its preferred equilibrium on the strategy A. Nevertheless, for UI in the diagonal case there is a minimal value $\Delta S \approx 2$ below which the Pareto-optimal phase does not exist at all, hence here the proximity of layers can not be the explanation of synchronisation in the payoff-dominant equilibrium. Moreover, there is an optimal size of the gap $\Delta S$ for which the Pareto-optimal phase is the widest. For UI it is approximately the maximal gap $\Delta S_{max}$ and for RD it is one of the middle values, but certainly not the smallest gap. These considerations lead us to a conclusion that synchronisation and equilibrium selection in coordination games on multilayer networks are very complex phenomena where obtaining the most advantageous outcome requires accurate parameter selection.

## Discussion

We investigated synchronisation between layers and equilibrium selection in the general coordination game on a multilayer network. The game played on each layer is described by a different payoff matrix, but both games are equally distant from the risk-dominance transition line $T=S+1$. The layers are connected by *Nq* inter-links, where the parameter *q* is the node overlap or degree of multiplexity. We studied the impact of of the value of *q* and the gap $\Delta S$ between the layers for three update rules: the replicator dynamics, the best response, and the unconditional imitation.

The most prominent outcome is the symmetry breaking in equilibrium selection. In neither of the cases, diagonal and stag hunt, there is a difference in average payoffs of games played on the layers. The strategies preferred by each layer are equally risk-dominant, i.e. the distance from the transition line $T=S+1$ is the same. The only difference, of course, is that the strategy A gives the highest possible payoff, hence it’s the most profitable one. A common-sense approach would lead us to believe that the payoff-dominant strategy A should be naturally promoted by the population. This is however not the case on single-layer networks, where the risk-dominant strategy is always selected in the range of connectivities that we considered^19^. In our multilayer model, which strategy is risk-dominant depends on the layer, but coordination on the strategy A prevails in most of the parameters space or is at least favoured on average. It is therefore clear that the multilayer structure enhances the Pareto-optimal outcome and it does so in a complex manner.

We identified three main phases depending on the node overlap *q* and the gap size $\Delta S$. The first one for lower values of *q* is a no-synchronisation phase, where each layer obtains a certain level of coordination close to its preferred equilibrium ($\alpha ^I \ne \alpha ^{II}$). The second phase begins when layers become synchronised (i.e. $\Delta \alpha$ drops to zero) and fully coordinate on the Pareto-optimal strategy A. Finally, the third phase appears for the highest node overlap. In this phase layers are also synchronised and they also coordinate, but not always on the strategy A—either equilibrium is possible, although depending on the parameters one of them might be preferred on average. In some cases the second phase does not appear.

The Pareto-optimal phase is not a mere effect of high node overlap between layers or low gap size. It has a more complex shape that depends on both parameters and on the update rule. For BR the Pareto-optimal phase does not exist at all. For RD it is placed, surprisingly, in the middle rage of the node overlap *q*, but its position and width depend also on $\Delta S$. Neither too low nor too high degree of multiplexity helps in achieving the optimal equilibrium, and the same is true for the gap size. Nevertheless, the value of $q_c$ grows with increasing distance $\Delta S$. For UI the Pareto-optimal phase might not even exist for lower values of $\Delta S$. If the phase exists, however, it appears already for any non-zero node overlap, as the synchronisation is much faster for UI.

Our work contributes to the understanding of equilibrium selection in coordination games, bringing in the general context of multilayer networks. Since many socio-technical systems have multiple environments where people can interact, the application of layered structures in their modelling is a natural step forward. As we showed, this approach can be highly relevant in analysis of coordination dilemmas, because it leads to non-trivial new effects that have not been observed in single-layer networks.

## Methods

We run numerical simulations of the general coordination game defined by the payoff matrix (1) on a multilayer graph. Agents are placed on two networks of *N* nodes forming two layers of the multilayer network. Each layer is a random regular graph with a degree *k*, generated using $\texttt {K}\_\texttt {Regular}$, algorithm form the *igraph* python package^68,69^. The coupling between layers can be adjusted using two parameters: node overlap *q* and edge overlap. As we didn’t observe any influence of varying edge overlap on the results we maintain a perfect edge overlap, i.e. both layers have exactly the same structure of connections. The node overlap *q* takes values from 0 to 1, defining the fraction of nodes connected (or shared) between both layers. At the beginning of each simulation every node on each layer is assigned a strategy A or B with equal probability, what gives the initial level of $\alpha = 0.5$. If two nodes are shared, their state has to be the same on both layers at all times. In other words, it’s the same node present on both layers. For $q=0$ there is no connection between the layers and their dynamics are fully separated, for $q=1$ it’s effectively a single-layer network with each game played half of the time.

The game played on each layer is described by different values of $S^\beta$ and $T^\beta$ parameters of the payoff matrix, given in Eq. (2). We use an asynchronous algorithm where at the beginning of each time step a layer is randomly selected with equal probability for both layers. Then, the update is performed on the chosen layer as for a single-layer network and according to the game played on the layer. First, a random node is chosen with equal probability for all nodes on the layer. We call it the active or focal node. The active node then plays the game with all its *k* neighbours on the layer and receives a given payoff, which is saved. Finally, the strategy of the active node is updated according to one of the following three update rules:

- the Replicator Dynamics (RD) (aka replicator rule, or proportional imitation rule)—the active node compares the payoff with a random neighbour on the layer and copies its strategy with probability $p=\frac{\mathrm {payoff~difference}}{k(1-\textrm{min}\{T^I,S^I, T^{II},S^{II}\})}$, if the neighbour’s payoff is bigger. The denominator is equal to the largest possible payoff difference allowed by the payoff matrix and network structure and it sets the probability *p* within [0, 1] range. Note, that we use the global maximal (1) and minimal ($\textrm{min}\{T^I,S^I, T^{II},S^{II}\}$) payoffs in order to keep the probability of copying normalised, since nodes that obtained their payoffs on different layers can interact with each other,
- the myopic Best Response (BR)—the active node chooses the best strategy given the current strategies of the neighbours on the layer, i.e. it compares all payoffs it would obtain playing each possible strategy against the current strategies of the neighbours and chooses the strategy resulting in the largest payoff,
- the Unconditional Imitation (UI)—the active node copies the strategy of the most successful neighbour on the layer, i.e. the one with the highest payoff, if its payoff is bigger.

At the end, the state of the focal node is copied onto the other layer, if the updated node is connected (shared) between the layers. More precisely, the new strategy selected by the node and the last payoff are copied. The simulation runs until a stationary state is reached, or a frozen configuration is obtained on all layers.

## Supplementary Information

Supplementary Information.

## Data Availability

This is a computational study and the only data considered is the result of simulations of the coordination game. To reproduce the research one should recreate the simulations. We facilitate this by describing in detail the model and tools used in the main text. The datasets generated during the current study are available from the corresponding author on reasonable request.
