# Supplementary material for: Evolutionary games on multilayer networks: coordination and equilibrium selection
Source: Sci Rep. 2023 Jul 21;13:11818. doi: 10.1038/s41598-023-38589-6 (PMC10362047; doi:10.1038/s41598-023-38589-6)
Supplement: Supplementary file 1 — Supplementary Information. [file 41598_2023_38589_MOESM1_ESM.pdf]

Supplementary material for:
Evolutionary games on multilayer networks: coordination and equilibrium selection

Tomasz Raducha

tjan@math.uc3m.es

Grupo Interdisciplinar de Sistemas Complejos (GISC),

Departamento de Matemáticas, Universidad Carlos III de Madrid, Leganés, Spain

Instituto de Física Interdisciplinar y Sistemas Complejos IFISC (CSIC-UIB), Palma, Spain

Maxi San Miguel

Instituto de Física Interdisciplinar y Sistemas Complejos IFISC (CSIC-UIB), Palma, Spain

In this supplementary material we provide additional plots of coordination rate α for $q = 1$ (as in Figure 2 from the main manuscript), plots of coordination rate α^I and α^{II} on each layer for varying q (as in Figure 3 from the main manuscript), plots of the dependence of q_c on the gap ΔS or ΔT (as in Figure 4 from the main manuscript), and phase diagrams showing the dependence of synchronisation and coordination on q and ΔS or ΔT (as in Figure 6 from the main manuscript). In addition to the cases considered in the main text we show results for *horizontal* and *vertical* cases, which are depicted in Supplementary Figure S1.

For the horizontal case all points lay on the line $T^\beta = -1$ and for the vertical case on $S^\beta = -2$, $\beta \in \{\text{I, II}\}$. Since the layers are placed symmetrically around the $T = S + 1$ line, or more precisely around a point (S_0, T_0) at this line, the parameter $\Delta S = S^I - S^{II}$ or $\Delta T = T^I - T^{II}$ is sufficient to determine values of all four parameters S^I, T^I, S^{II}, T^{II}. Namely:

$$
\begin{aligned}
S_h^I &= S_0 + \frac{\Delta S}{2}, \\
T_h^I &= -1, \\
S_h^{II} &= S_0 - \frac{\Delta S}{2}, \\
T_h^{II} &= -1,
\end{aligned}
\tag{1}
$$

for the horizontal case and:

$$
\begin{aligned}
S_v^I &= -2, \\
T_v^I &= T_0 - \frac{\Delta T}{2}, \\
S_v^{II} &= -2, \\
T_v^{II} &= T_0 + \frac{\Delta T}{2},
\end{aligned}
\tag{2}
$$

for the vertical case, where $(S_0, T_0) = (-2, -1)$. Note, that horizontal and vertical cases are not fully symmetrical in respect to the games played on the layers. In the horizontal case the game played on layer I has always higher average payoff and in the vertical case the average payoff is bigger on layer II.

It is worth mentioning that results for BR for higher connectivities indicate that bigger degree may destroy synchronisation (Supplementary Figures S8, S10, and S12). Synchronisation is also weakened in the horizontal case for RD when changing the connectivity from $k = 8$ to a complete graph (Supplementary Figure S5). Additionally, it seems to be detrimental for the Pareto-optimal equilibrium. It is especially interesting, since the Pareto-optimal phase is abundant for UI on a complete graph, except for the vertical case in which the layer II has higher average payoff (Supplementary Figures S14-S17). Finally, the Pareto-optimal phase and synchronisation region are bigger in the vertical case than in the horizontal case for RD (Supplementary Figures S4 and S6). This is especially surprising, because the vertical case is biased towards layer II, as mentioned before, and the horizontal case favours layer I (in terms of the average payoff). The horizontal case results in a bigger Pareto-optimal phase for UI, but surprisingly the effect is inverted for RD.

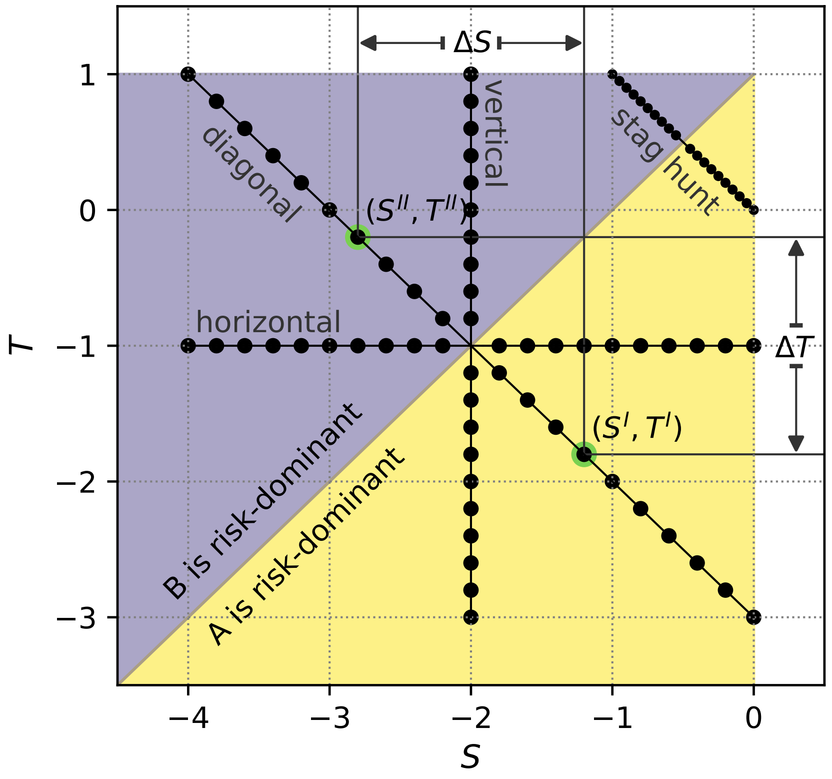

Supplementary Figure S1: Diagram of the S-T parameter space showing parametrisation of the layers. Each circle on the diagonal lines represents a game played on one of the layers. On layer I the strategy A is always risk-dominant (yellow area), and on layer II the strategy B is always risk-dominant (purpule area). Risk-dominance changes at the line $T = S + 1$. Examplary values of (S^I, T^I) and (S^{II}, T^{II}) are highlighted in green with ΔS and ΔT illustrated.

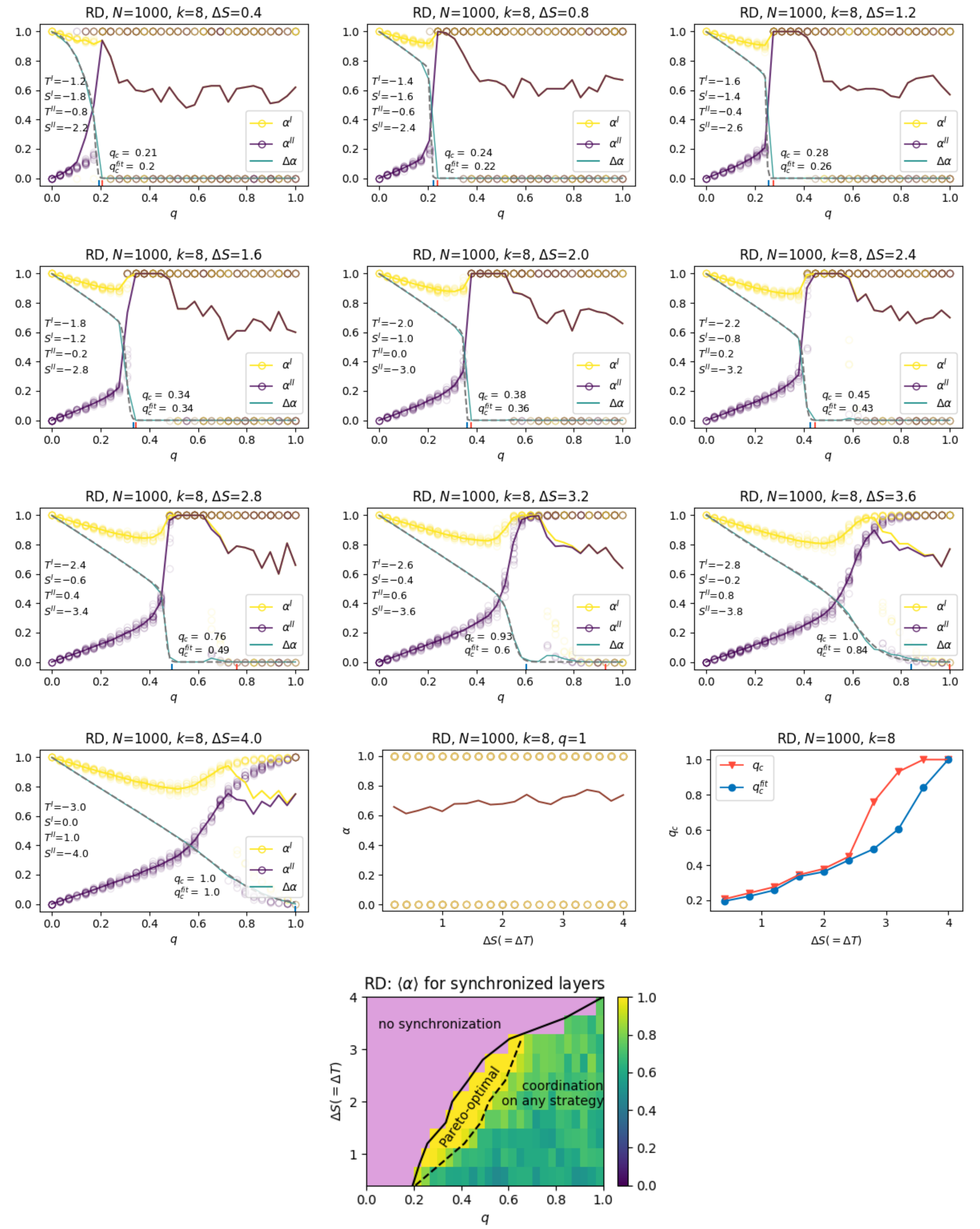

Supplementary Figure S2: **RD in the diagonal case** (i) Coordination rates on layers α^I, α^{II}, and $\Delta\alpha$ vs node overlap q for all values of ΔS. (ii) Coordination rate $\alpha = \alpha^I = \alpha^{II}$ vs gap size ΔS for full node overlap $q = 1$. (iii) Critical value of q_c and q_c^{fit} vs gap size ΔS. (iv) Phase diagram of coordination rate $\alpha = \alpha^I = \alpha^{II}$ in the q-ΔS space for synchronised layers. Each layer has $N = 1000$ nodes with an intra-layer degree $k = 8$. Averaged over (at least) 100 realisations.

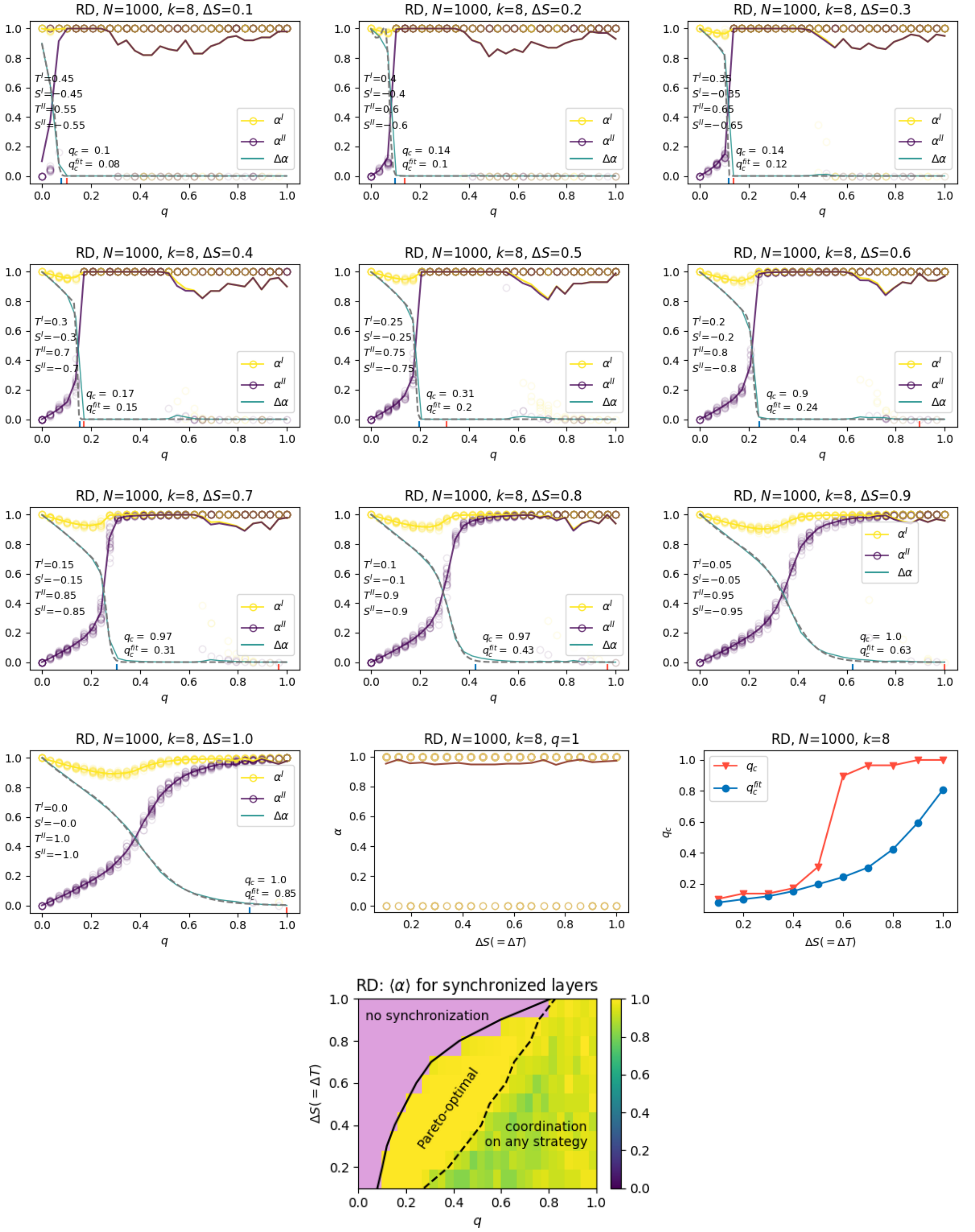

Supplementary Figure S3: **RD in the stag hunt case** (i) Coordination rates on layers α^I, α^{II}, and $\Delta\alpha$ vs node overlap q for all values of ΔS. (ii) Coordination rate $\alpha = \alpha^I = \alpha^{II}$ vs gap size ΔS for full node overlap $q = 1$. (iii) Critical value of q_c and q_c^{fit} vs gap size ΔS. (iv) Phase diagram of coordination rate $\alpha = \alpha^I = \alpha^{II}$ in the q-ΔS space for synchronised layers. Each layer has $N = 1000$ nodes with an intra-layer degree $k = 8$. Averaged over (at least) 100 realisations.

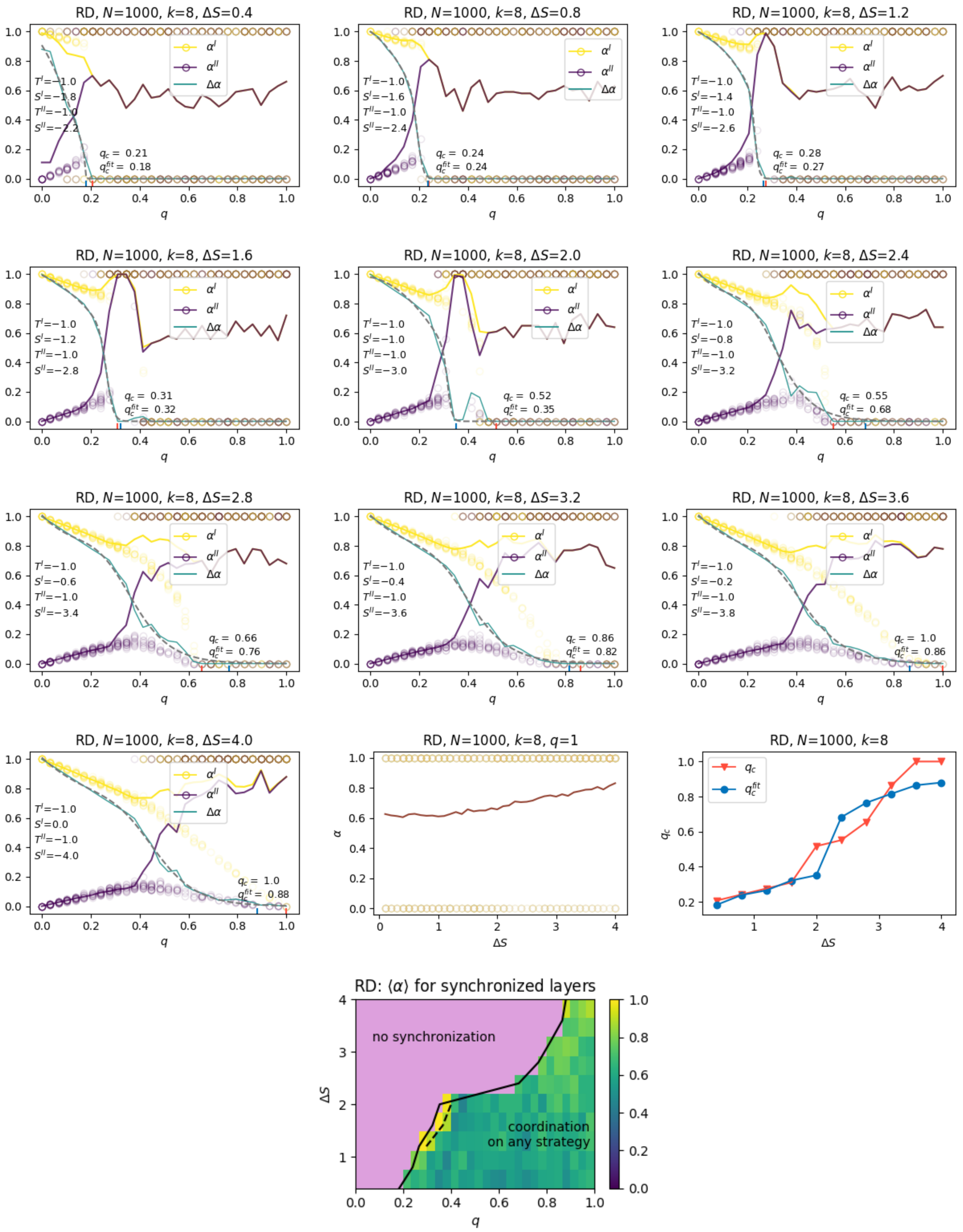

Supplementary Figure S4: **RD in the horizontal case** (i) Coordination rates on layers α^I, α^{II}, and $\Delta\alpha$ vs node overlap q for all values of ΔS. (ii) Coordination rate $\alpha = \alpha^I = \alpha^{II}$ vs gap size ΔS for full node overlap $q = 1$. (iii) Critical value of q_c and q_c^{fit} vs gap size ΔS. (iv) Phase diagram of coordination rate $\alpha = \alpha^I = \alpha^{II}$ in the q-ΔS space for synchronised layers. Each layer has $N = 1000$ nodes with an intra-layer degree $k = 8$. Averaged over (at least) 100 realisations.

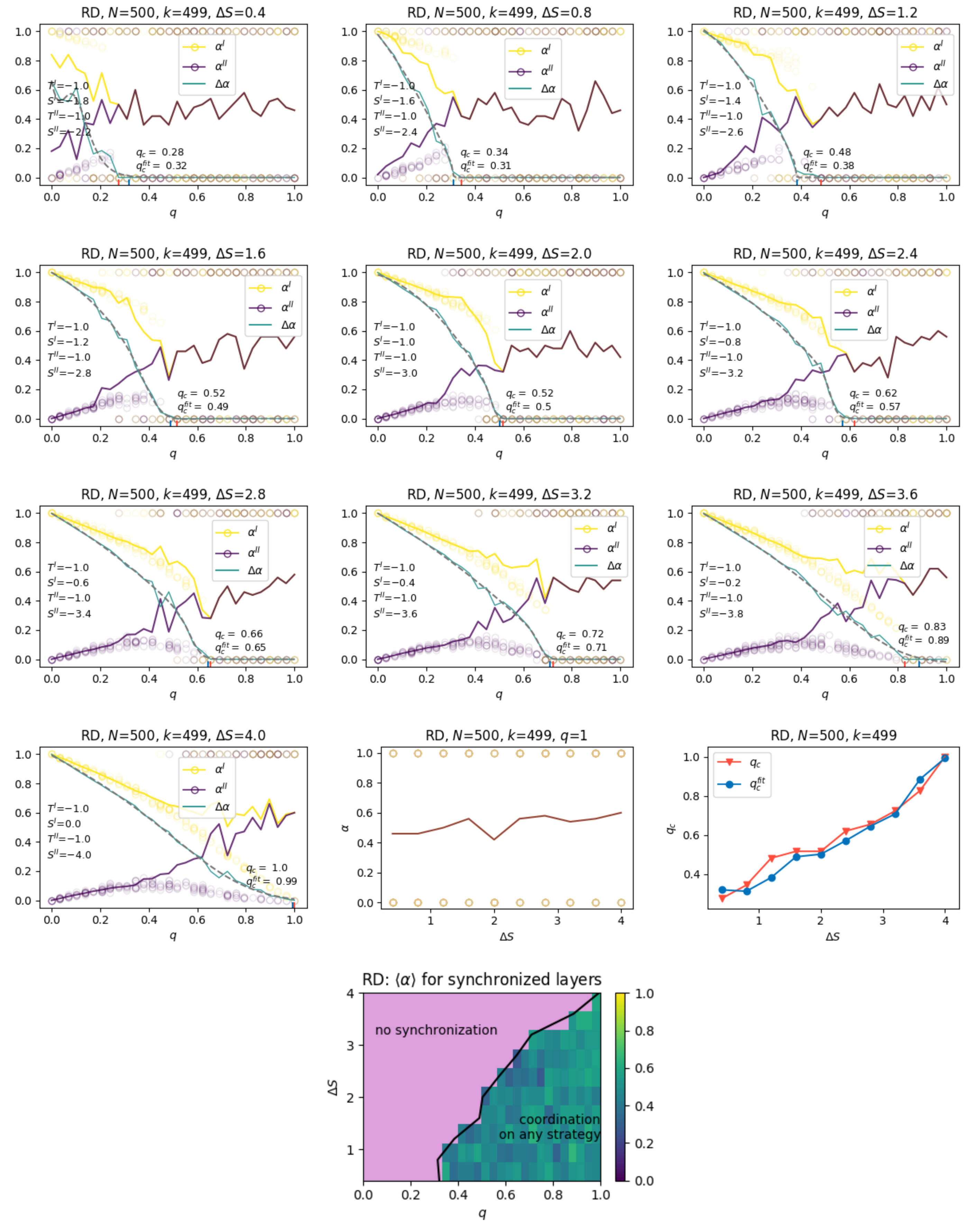

Supplementary Figure S5: **RD in the horizontal case** (i) Coordination rates on layers α^I, α^{II}, and $\Delta\alpha$ vs node overlap q for all values of ΔS. (ii) Coordination rate $\alpha = \alpha^I = \alpha^{II}$ vs gap size ΔS for full node overlap $q = 1$. (iii) Critical value of q_c and q_c^{fit} vs gap size ΔS. (iv) Phase diagram of coordination rate $\alpha = \alpha^I = \alpha^{II}$ in the q-ΔS space for synchronised layers. Each layer has $N = 500$ nodes and forms a complete graph. Averaged over (at least) 50 realisations.

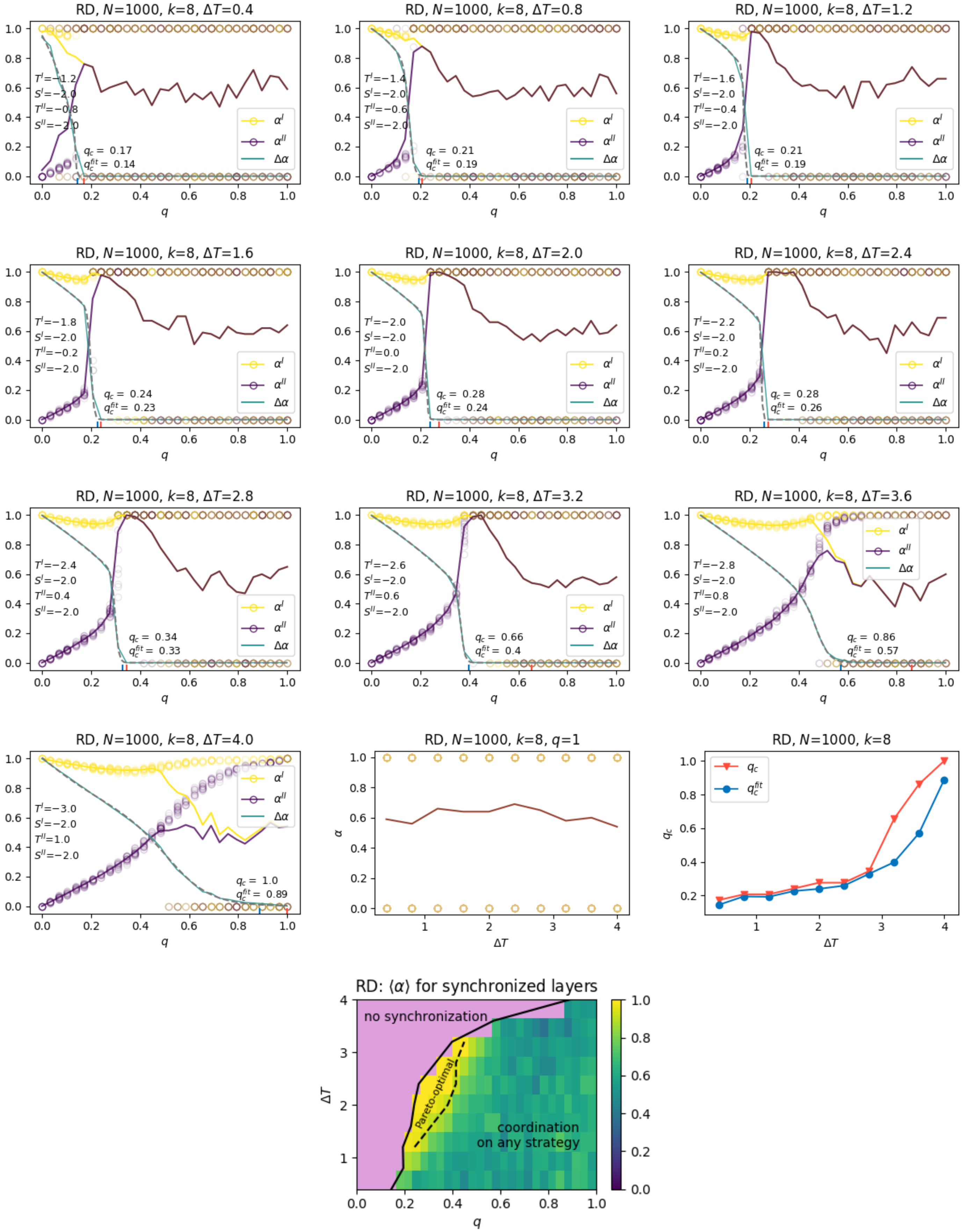

Supplementary Figure S6: **RD in the vertical case** (i) Coordination rates on layers α^I, α^{II}, and $\Delta\alpha$ vs node overlap q for all values of ΔT. (ii) Coordination rate $\alpha = \alpha^I = \alpha^{II}$ vs gap size ΔT for full node overlap $q = 1$. (iii) Critical value of q_c and q_c^{fit} vs gap size ΔT. (iv) Phase diagram of coordination rate $\alpha = \alpha^I = \alpha^{II}$ in the q-ΔT space for synchronised layers. Each layer has $N = 1000$ nodes with an intra-layer degree $k = 8$. Averaged over (at least) 100 realisations.

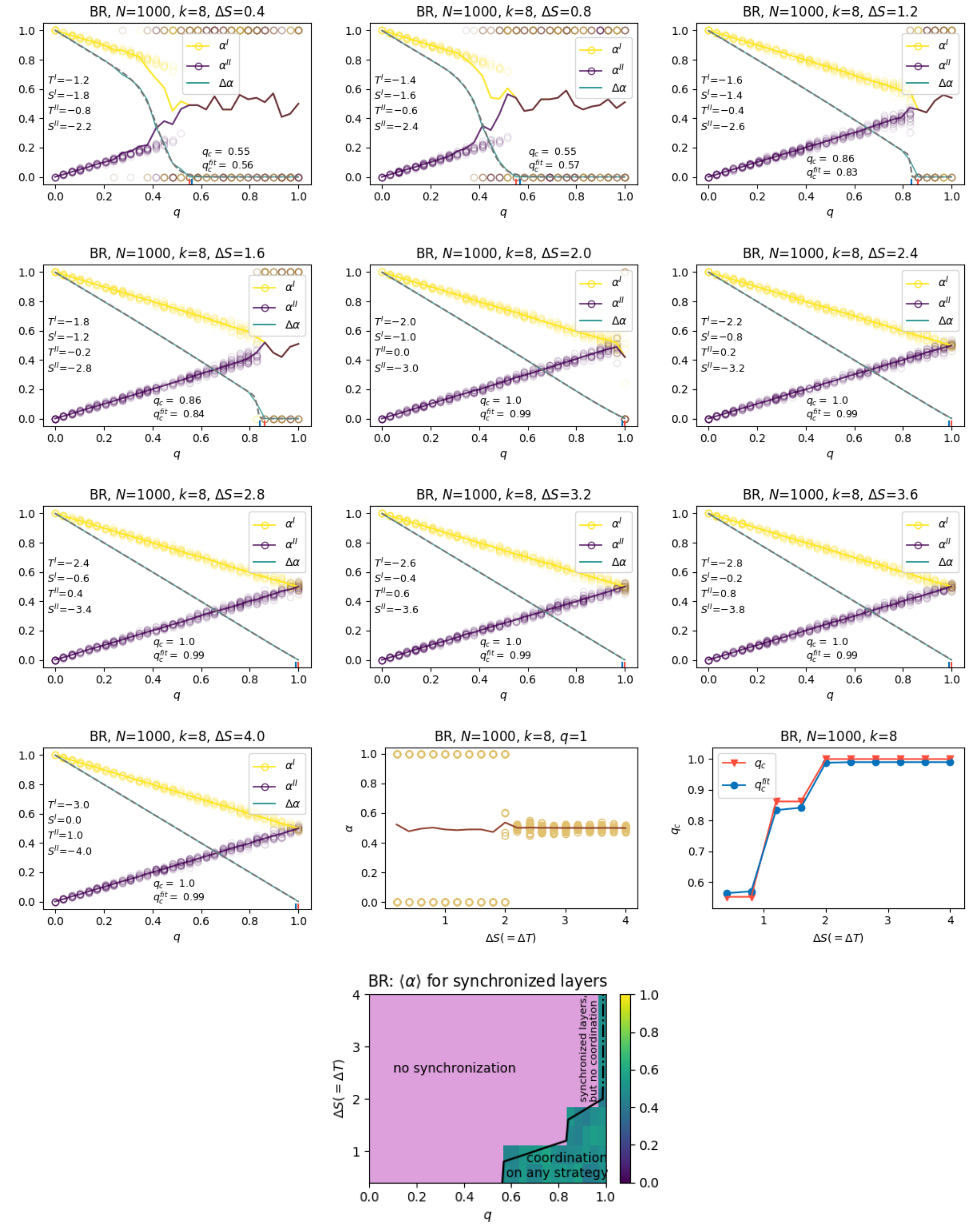

Supplementary Figure S7: **BR in the diagonal case** (i) Coordination rates on layers α^I, α^{II}, and $\Delta\alpha$ vs node overlap q for all values of ΔS. (ii) Coordination rate $\alpha = \alpha^I = \alpha^{II}$ vs gap size ΔS for full node overlap $q = 1$. (iii) Critical value of q_c and q_c^{fit} vs gap size ΔS. (iv) Phase diagram of coordination rate $\alpha = \alpha^I = \alpha^{II}$ in the q-ΔS space for synchronised layers. Each layer has $N = 1000$ nodes with an intra-layer degree $k = 8$. Averaged over (at least) 100 realisations.

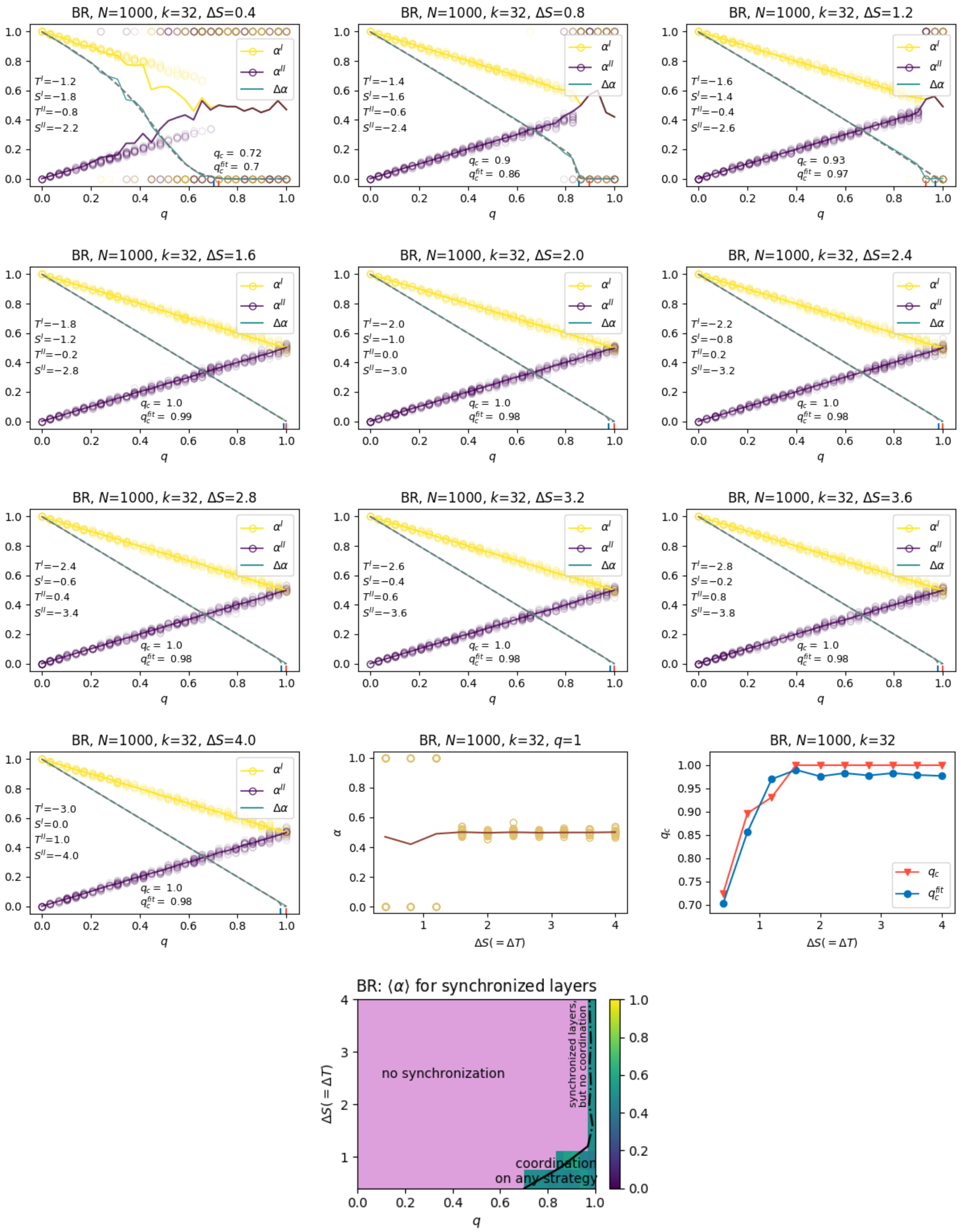

Supplementary Figure S8: **BR in the diagonal case** (i) Coordination rates on layers α^I, α^{II}, and $\Delta\alpha$ vs node overlap q for all values of ΔS. (ii) Coordination rate $\alpha = \alpha^I = \alpha^{II}$ vs gap size ΔS for full node overlap $q = 1$. (iii) Critical value of q_c and q_c^{fit} vs gap size ΔS. (iv) Phase diagram of coordination rate $\alpha = \alpha^I = \alpha^{II}$ in the q-ΔS space for synchronised layers. Each layer has $N = 1000$ nodes with an intra-layer degree $k = 32$. Averaged over (at least) 100 realisations.

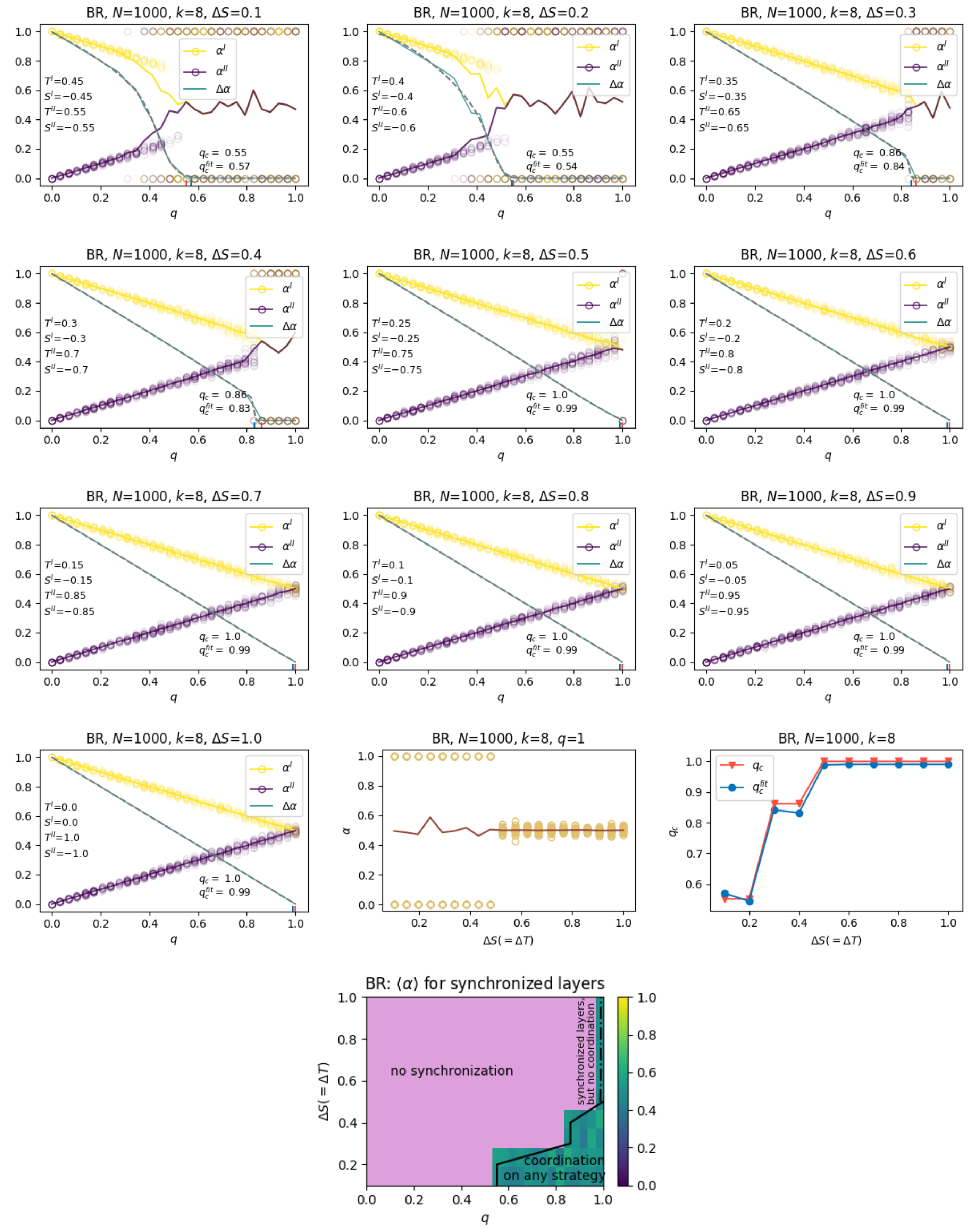

Supplementary Figure S9: **BR in the stag hunt case** (i) Coordination rates on layers α^I, α^{II}, and $\Delta\alpha$ vs node overlap q for all values of ΔS. (ii) Coordination rate $\alpha = \alpha^I = \alpha^{II}$ vs gap size ΔS for full node overlap $q = 1$. (iii) Critical value of q_c and q_c^{fit} vs gap size ΔS. (iv) Phase diagram of coordination rate $\alpha = \alpha^I = \alpha^{II}$ in the q-ΔS space for synchronised layers. Each layer has $N = 1000$ nodes with an intra-layer degree $k = 8$. Averaged over (at least) 100 realisations.

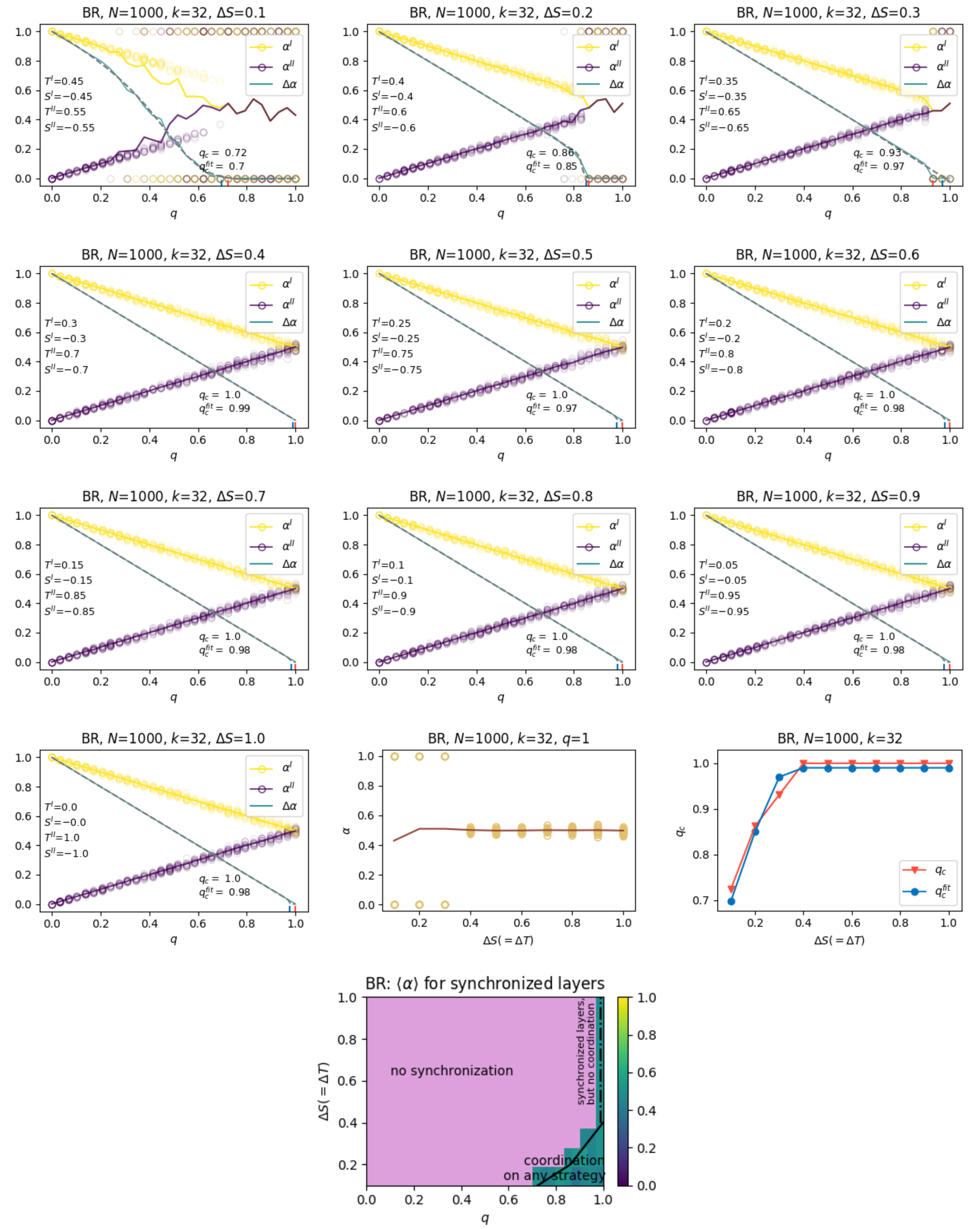

Supplementary Figure S10: **BR in the stag hunt case** (i) Coordination rates on layers α^I, α^{II}, and $\Delta\alpha$ vs node overlap q for all values of ΔS. (ii) Coordination rate $\alpha = \alpha^I = \alpha^{II}$ vs gap size ΔS for full node overlap $q = 1$. (iii) Critical value of q_c and q_c^{fit} vs gap size ΔS. (iv) Phase diagram of coordination rate $\alpha = \alpha^I = \alpha^{II}$ in the q-ΔS space for synchronised layers. Each layer has $N = 1000$ nodes with an intra-layer degree $k = 32$. Averaged over (at least) 100 realisations.

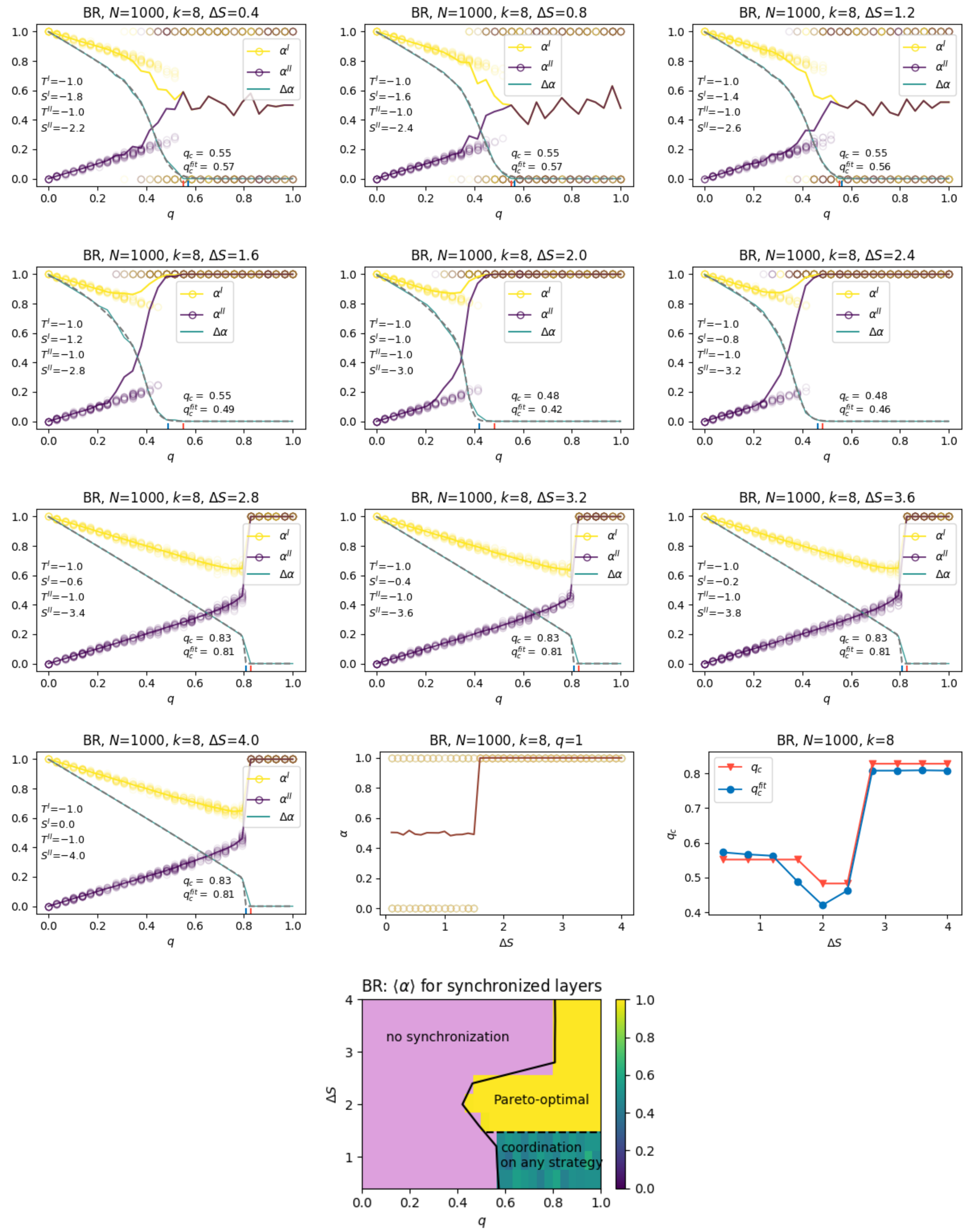

Supplementary Figure S11: **BR in the horizontal case** (i) Coordination rates on layers α^I, α^{II}, and $\Delta\alpha$ vs node overlap q for all values of ΔS. (ii) Coordination rate $\alpha = \alpha^I = \alpha^{II}$ vs gap size ΔS for full node overlap $q = 1$. (iii) Critical value of q_c and q_c^{fit} vs gap size ΔS. (iv) Phase diagram of coordination rate $\alpha = \alpha^I = \alpha^{II}$ in the q-ΔS space for synchronised layers. Each layer has $N = 1000$ nodes with an intra-layer degree $k = 8$. Averaged over (at least) 100 realisations.

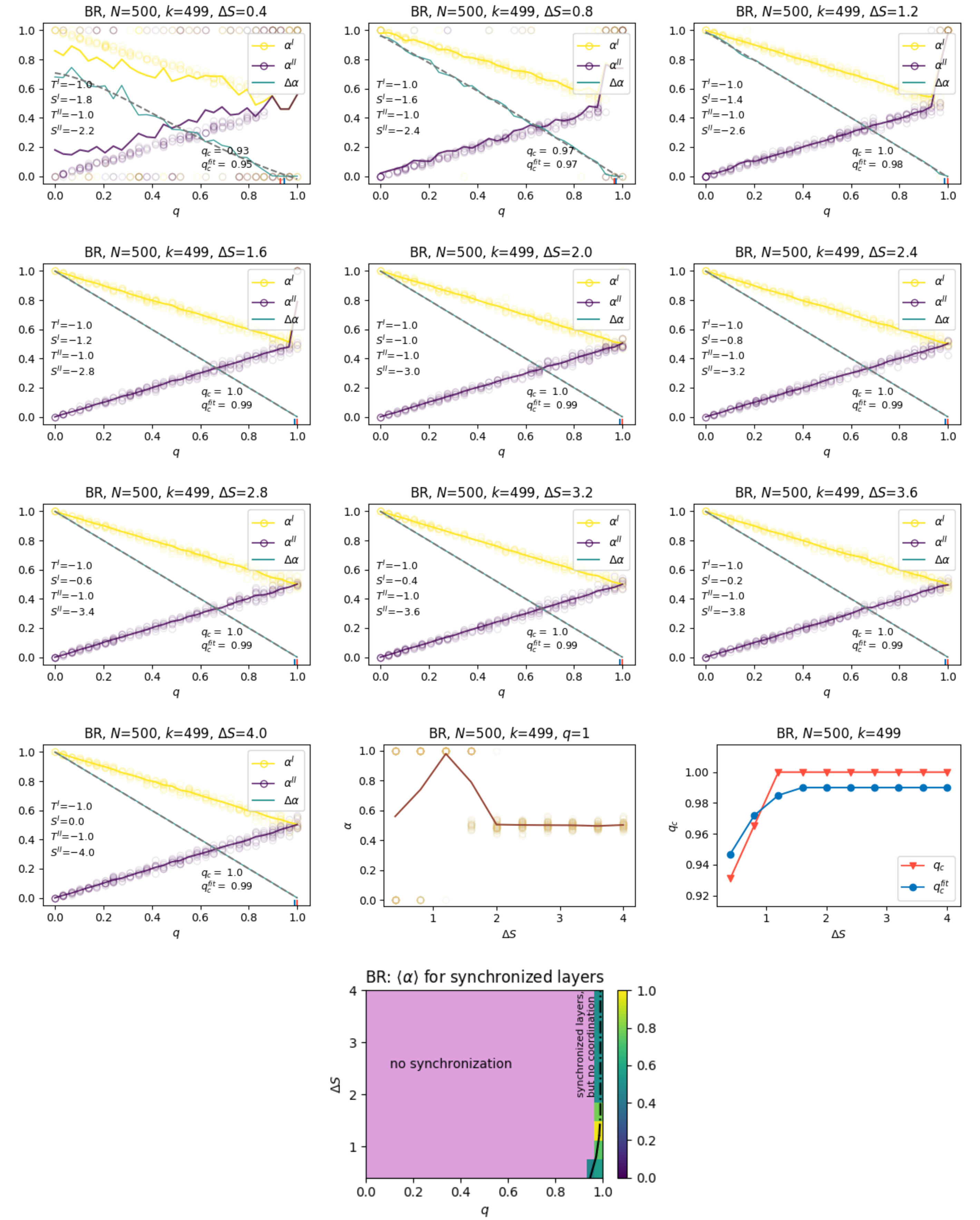

Supplementary Figure S12: **BR in the horizontal case** (i) Coordination rates on layers α^I, α^{II}, and $\Delta\alpha$ vs node overlap q for all values of ΔS. (ii) Coordination rate $\alpha = \alpha^I = \alpha^{II}$ vs gap size ΔS for full node overlap $q = 1$. (iii) Critical value of q_c and q_c^{fit} vs gap size ΔS. (iv) Phase diagram of coordination rate $\alpha = \alpha^I = \alpha^{II}$ in the q-ΔS space for synchronised layers. Each layer has $N = 500$ nodes and forms a complete graph. Averaged over (at least) 50 realisations.

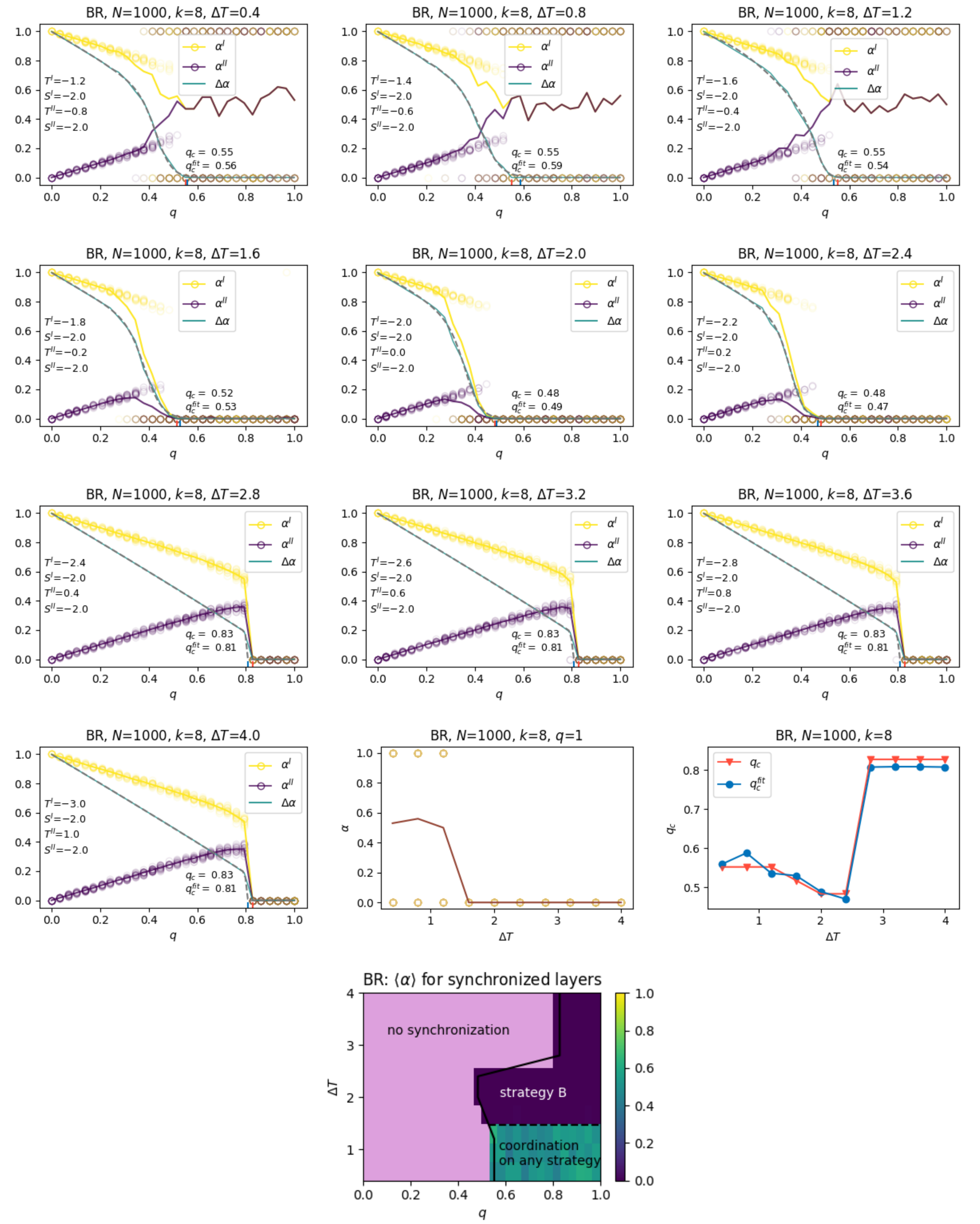

Supplementary Figure S13: **BR in the vertical case** (i) Coordination rates on layers α^I, α^{II}, and $\Delta\alpha$ vs node overlap q for all values of ΔT. (ii) Coordination rate $\alpha = \alpha^I = \alpha^{II}$ vs gap size ΔT for full node overlap $q = 1$. (iii) Critical value of q_c and q_c^{fit} vs gap size ΔT. (iv) Phase diagram of coordination rate $\alpha = \alpha^I = \alpha^{II}$ in the q-ΔT space for synchronised layers. Each layer has $N = 1000$ nodes with an intra-layer degree $k = 8$. Averaged over (at least) 100 realisations.

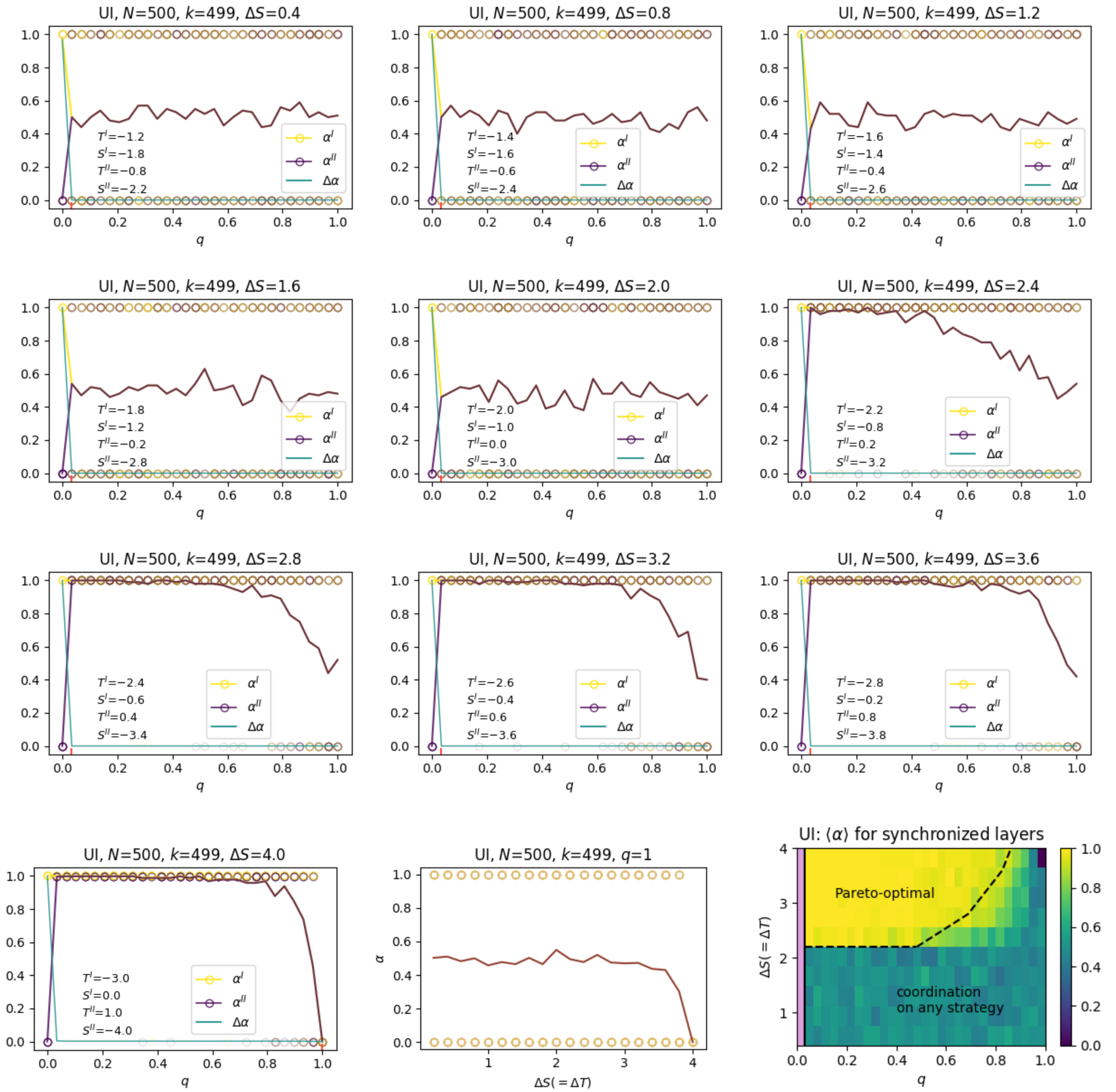

Supplementary Figure S14: **UI in the diagonal case** (i) Coordination rates on layers α^I, α^{II}, and $\Delta\alpha$ vs node overlap q for all values of ΔS. (ii) Coordination rate $\alpha = \alpha^I = \alpha^{II}$ vs gap size ΔS for full node overlap $q = 1$. (iii) Phase diagram of coordination rate $\alpha = \alpha^I = \alpha^{II}$ in the q-ΔS space for synchronised layers. Each layer has $N = 1000$ nodes and forms a complete graph. Averaged over (at least) 100 realisations.

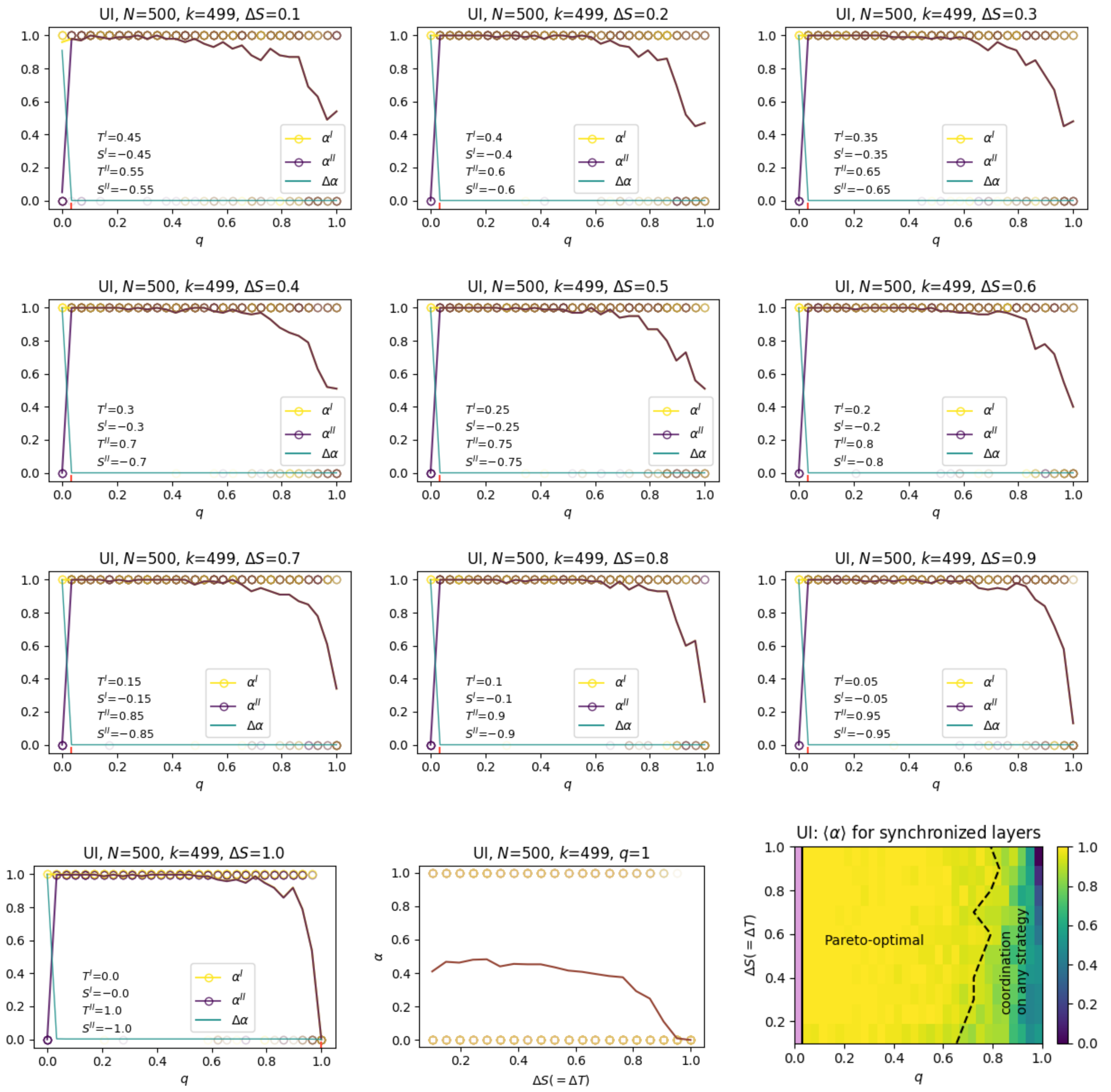

Supplementary Figure S15: **UI in the stag hunt case** (i) Coordination rates on layers α^I, α^{II}, and $\Delta\alpha$ vs node overlap q for all values of ΔS. (ii) Coordination rate $\alpha = \alpha^I = \alpha^{II}$ vs gap size ΔS for full node overlap $q = 1$. (iii) Phase diagram of coordination rate $\alpha = \alpha^I = \alpha^{II}$ in the q-ΔS space for synchronised layers. Each layer has $N = 1000$ nodes and forms a complete graph. Averaged over (at least) 100 realisations.

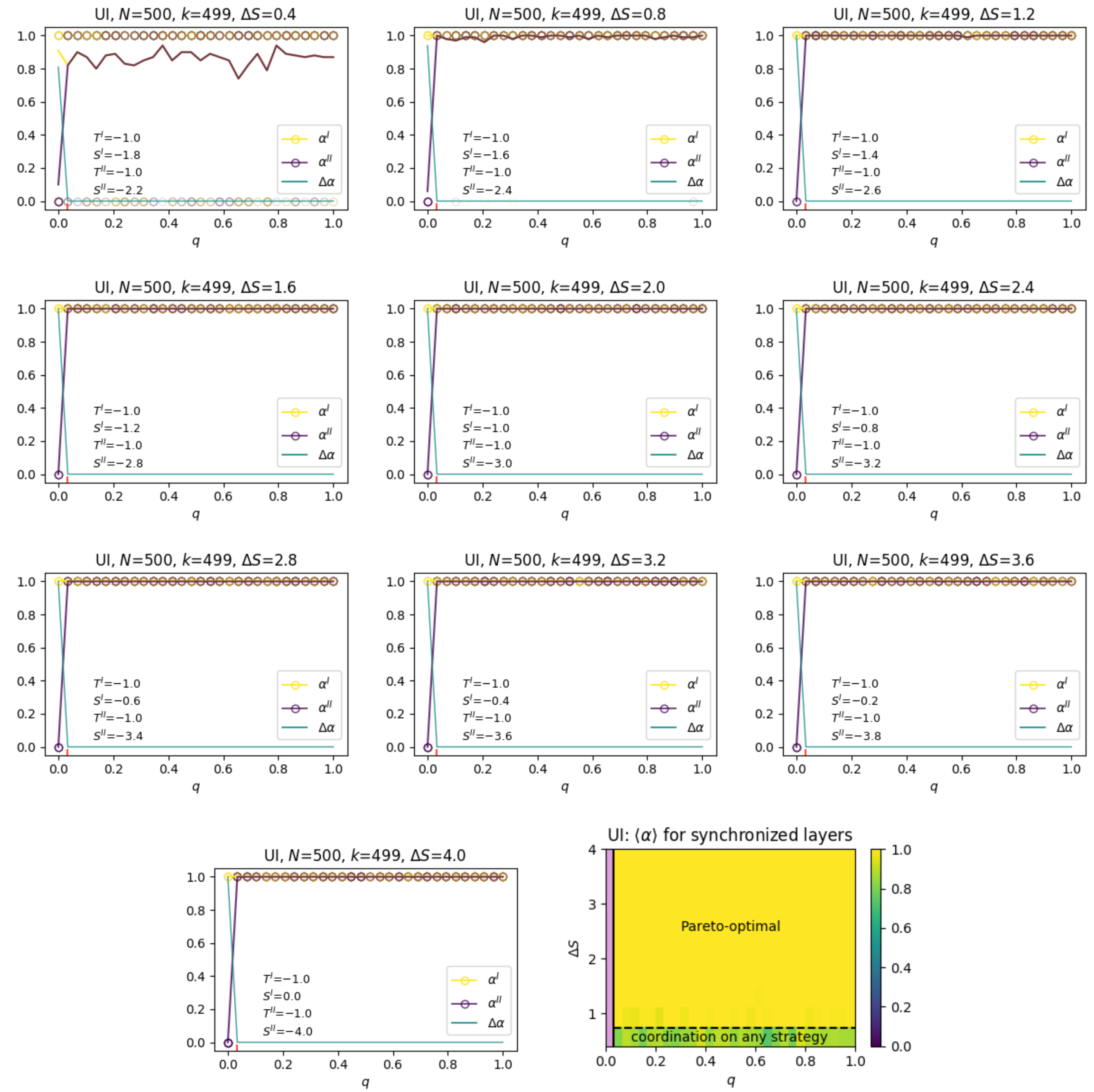

Supplementary Figure S16: **UI in the horizontal case** (i) Coordination rates on layers α^I, α^{II}, and $\Delta\alpha$ vs node overlap q for all values of ΔS. (ii) Phase diagram of coordination rate $\alpha = \alpha^I = \alpha^{II}$ in the q-ΔS space for synchronised layers. Each layer has $N = 1000$ nodes and forms a complete graph. Averaged over (at least) 100 realisations.

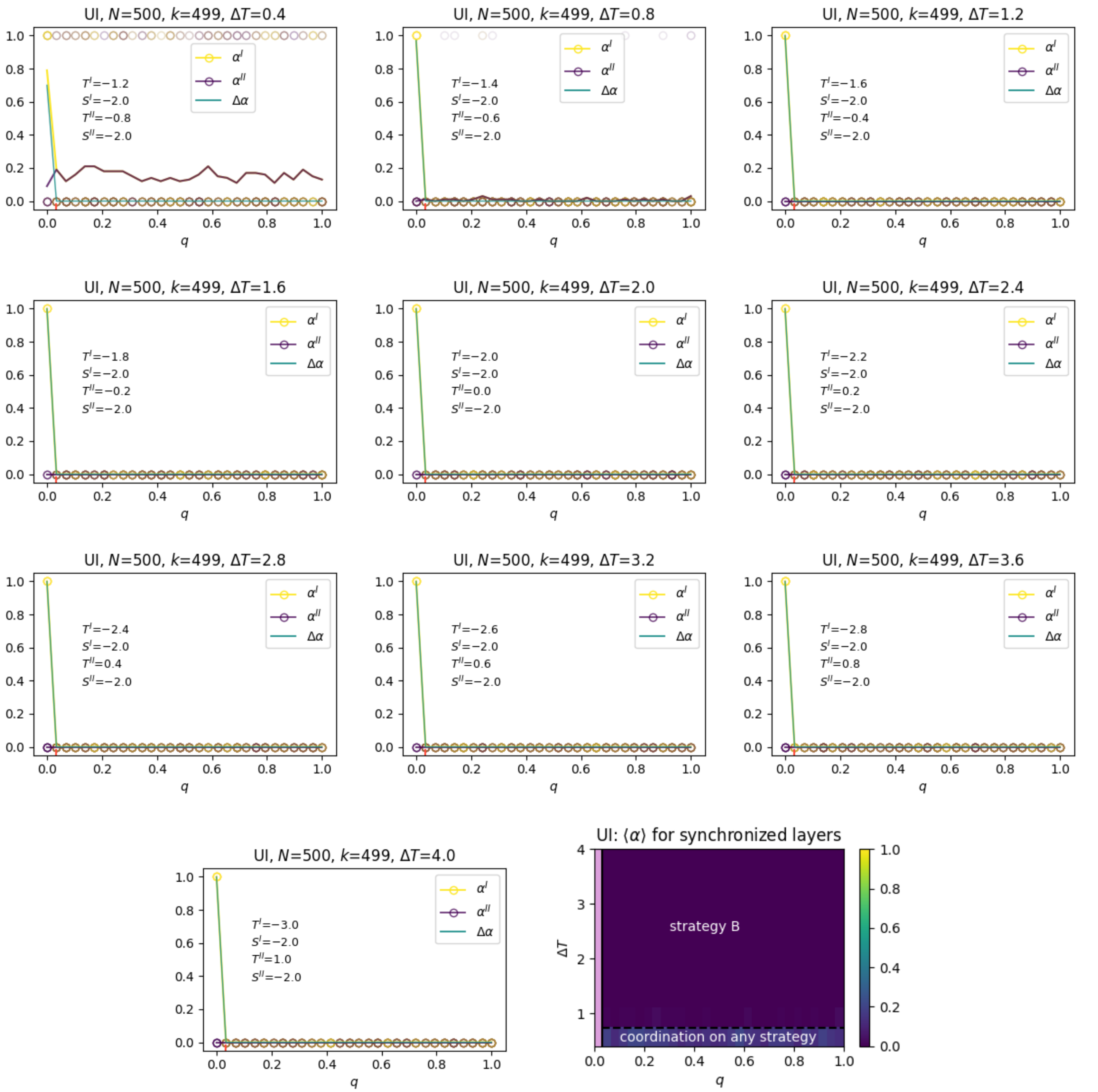

Supplementary Figure S17: **UI in the vertical case** (i) Coordination rates on layers α^I, α^{II}, and $\Delta\alpha$ vs node overlap q for all values of ΔT. (ii) Phase diagram of coordination rate $\alpha = \alpha^I = \alpha^{II}$ in the q-ΔT space for synchronised layers. Each layer has $N = 1000$ nodes and forms a complete graph. Averaged over (at least) 100 realisations.
